# Aerobic exercise elicits clinical adaptations in myotonic dystrophy type 1 patients independently of pathophysiological changes

**DOI:** 10.1172/JCI156125

**Published:** 2022-05-16

**Authors:** Andrew I. Mikhail, Peter L. Nagy, Katherine Manta, Nicholas Rouse, Alexander Manta, Sean Y. Ng, Michael F. Nagy, Paul Smith, Jian-Qiang Lu, Joshua P. Nederveen, Vladimir Ljubicic, Mark A. Tarnopolsky

**Affiliations:** 1 Department of Kinesiology, McMaster University, Hamilton, Ontario, Canada.; 2 Praxis Genomics LLC, Atlanta, Georgia, USA.; 3 Department of Pediatrics, McMaster University Children’s Hospital, Hamilton, Ontario, Canada.; 4 Department of Pathology and Molecular Medicine/Neuropathology, McMaster University, Hamilton, Ontario, Canada.; 5 Exerkine Corp., McMaster University Medical Center, Hamilton, Ontario, Canada.

**Keywords:** Cell Biology, Muscle Biology, Mitochondria, Neuromuscular disease, RNA processing

## Abstract

**Background:**

Myotonic dystrophy type 1 (DM1) is a complex life-limiting neuromuscular disorder characterized by severe skeletal muscle atrophy, weakness, and cardiorespiratory defects. Exercised DM1 mice exhibit numerous physiological benefits that are underpinned by reduced CUG foci and improved alternative splicing. However, the efficacy of physical activity in patients is unknown.

**Methods:**

Eleven genetically diagnosed DM1 patients were recruited to examine the extent to which 12 weeks of cycling can recuperate clinical and physiological metrics. Furthermore, we studied the underlying molecular mechanisms through which exercise elicits benefits in skeletal muscle of DM1 patients.

**RESULTS:**

DM1 was associated with impaired muscle function, fitness, and lung capacity. Cycling evoked several clinical, physical, and metabolic advantages in DM1 patients. We highlight that exercise-induced molecular and cellular alterations in patients do not conform with previously published data in murine models and propose a significant role of mitochondrial function in DM1 pathology. Finally, we discovered a subset of small nucleolar RNAs (snoRNAs) that correlated to indicators of disease severity.

**Conclusion:**

With no available cures, our data support the efficacy of exercise as a primary intervention to partially mitigate the clinical progression of DM1. Additionally, we provide evidence for the involvement of snoRNAs and other noncoding RNAs in DM1 pathophysiology.

**Trial registration:**

This trial was approved by the HiREB committee (no. 7901) and registered under ClinicalTrials.gov (NCT04187482).

**Funding:**

Neil and Leanne Petroff. Canadian Institutes of Health Research Foundation (no. 143325).

## Graphical abstract

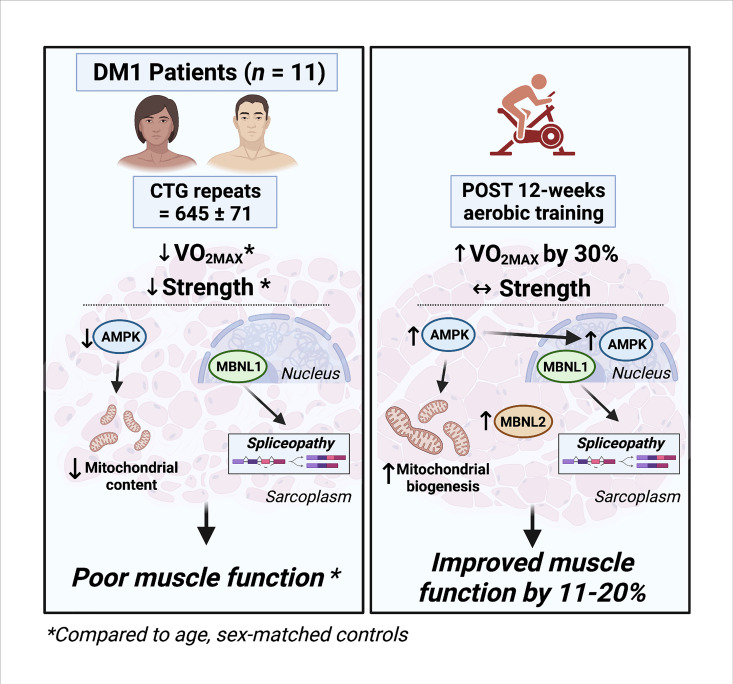

## Introduction

Myotonic dystrophy type 1 (DM1) is the most commonly diagnosed muscular dystrophy among adults and the second most prevalent of all muscular dystrophies (1). It is a progressive neuromuscular disorder (NMD) characterized by skeletal muscle wasting, weakness, and myotonia primarily in distal muscles of the upper extremities. There are often several other systemic defects, including cataracts, hypersomnolence, dysphagia, cardiac conduction block, gastrointestinal dysmotility, and endocrine disorders (2). DM1 is caused by an autosomal dominant microsatellite CTG repeat mutation in the 3′ UTR of the dystrophia myotonica protein kinase (*DMPK*) gene (3), which has been recently estimated to occur in approximately 1 in every 2100 births (4). Unaffected individuals have fewer than 40 repeats, while DM1 symptoms can manifest at between approximately 50 and approximately 3000 repeat expansions (3).

The microsatellite repeat expansion results in the nuclear accumulation of *DMPK* mRNA aggregates (5), triggering a dysregulation in the localization and activity of RNA-binding proteins (RNABPs) that are critical for splicing. Notably, the muscle-blind–like protein family (MBNL) has a high affinity for the “CUG” repetitive sequence, resulting in nuclear sequestration and reduced functional MBNL in the cytoplasm (6, 7). In contrast, CUG-binding protein 1 (CUGBP1/CELF1) functions antagonistically to MBNL, promoting the inclusion of fetal isoforms, and is hyperactivated in DM1 (8, 9). Collectively, the *DMPK* mRNA toxic gain-of-function disrupts MBNL and CUGBP1, resulting in a uniquely dysfunctional transcriptomic and splicing profile within skeletal muscle (10).

Efforts have been made during the past decade within the DM1 drug pipeline with several small molecules accelerating through preclinical and clinical stages; however, a decisive treatment for DM1 has not manifested. AMPK, a regulator of energy homeostasis (11), has gained attention as a promising therapeutic target against NMDs due to its emerging role in neuromuscular plasticity (12). Chronic stimulation of AMPK in DM1 mice corrected hallmark pathological features (13, 14), while in DM1 patients, 52 weeks of administration of metformin, a well-known AMPK activator, mildly improved mobility (15). Aerobic exercise is an inexpensive and safe intervention that can rapidly phosphorylate AMPK (16) and induce several physiological and molecular benefits in NMDs (17–21), but its efficacy in DM1 patients has yet to be fully elucidated. Previous studies suggested that aerobic training had no effect on muscle function (22, 23), while others reported increased aerobic capacity and myofiber size, but failed to measure functional or other physiological adaptations (24). Overall, the benefits of aerobic training at a physiological, cellular, and molecular level are largely unknown in this clinical population.

Herein, we show that 12 weeks of moderate intensity cycling elicits myriad benefits in DM1 patients without altering the molecular pathophysiology or transcriptomic signature relating to RNA toxicity. Furthermore, we highlight mitochondrial health as a central aspect of DM1 biology. Finally, we introduce small nucleolar RNAs (snoRNAs) as probable biomarkers for DM1 severity and potentially of diagnostic utility, which were further altered with exercise. Together, our data provide evidence to support the potential of exercise training to mitigate some clinical aspects of disease burden in DM1 patients.

## Results

### Patient characteristics, adherence, and safety.

**Figure 1:**
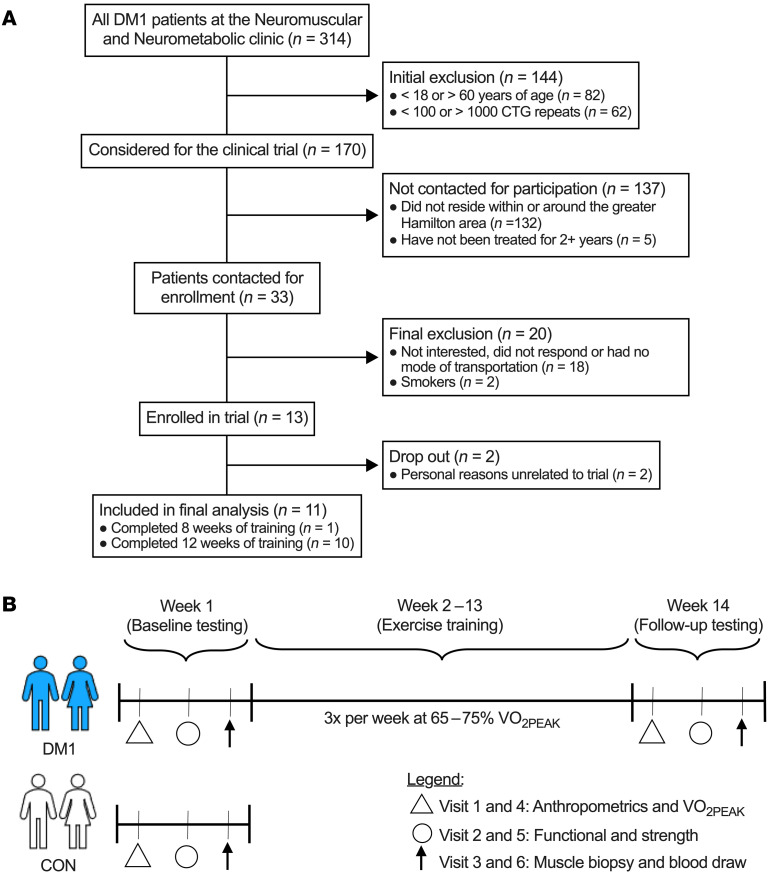
Flowchart of study enrollment and design. (**A**) CONSORT figure of the recruitment process. All DM1 patients at the Neuromuscular and Neurometabolic Clinic at McMaster University were considered for this trial. A total of 13 patients complied with the inclusion/exclusion criteria and were interested in participating. Eleven patients were included in the final analysis. (**B**) Brief schematic of the study design for DM1 patients and healthy CON. DM1 patients completed the full exercise trial (visits 1–6), while CON performed baseline testing only (visits 1–3) for reference values. Visits 1 and 4 consisted of anthropometric measures, body composition assessment, electrocardiography, and cardiorespiratory fitness assessment. Visits 2 and 5 included functional testing (6-MWT, 5XSTS, and TUG tests), spirometry testing, and strength testing (maximal isometric knee extension, grip strength, and pinch grip). Finally, participants reported fasting to the laboratory for visits 3 and 6 for a blood draw and a skeletal muscle biopsy from the vastus lateralis.

**Figure 2:**
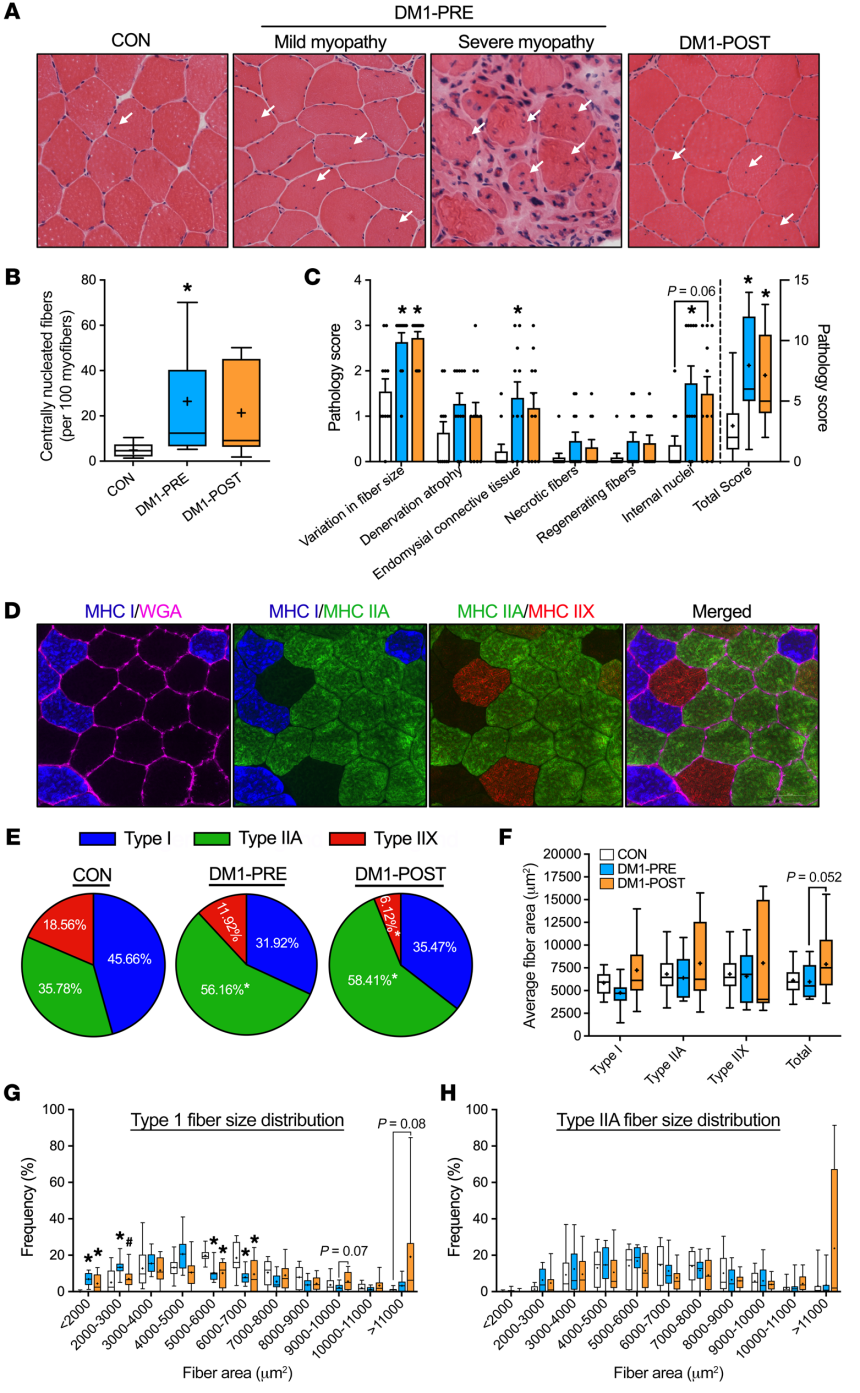
Exercise does not exacerbate myopathy and augments myofiber size in DM1 patients. (**A**) Representative images of H&E staining of the vastus lateralis muscle from CON (left) and DM1 patients before exercise with mild myopathy (DM1-PRE, middle left), DM1-PRE with severe myopathy (middle right), and patients following exercise (DM1-POST, right). Arrows indicate CNF. (**B**) Graphical summary of the frequency of CNF. (**C**) Average scores of pathology examination for CON, DM1-PRE, and DM1-POST. (**D**) Representative images of IF staining for wheat germ agglutinin (WGA) (cyan) and MHC type I (blue), type IIA (green), and type IIX (red). Original magnification, ×20. (**E**) Pie charts of fiber-type distribution in each group. (**F**) Average fiber-type–specific and total CSA. (**G** and **H**) Size distribution of fiber CSA for type I and type IIA fibers, respectively. Data are expressed as box and whisker plots with plus signs representing the mean (**B** and **F**–**H**) or bar graphs as mean ± SEM (**C**). *n* = 11. *P < 0.05 versus CON, 1-way ANOVA followed by Bonferroni’s correction; ^#^*P* < 0.05 versus DM1-PRE, 2-tailed paired *t* test corrected for multiple comparisons.

By design, DM1 patients before exercise (DM1-PRE) and control (CON) groups did not statistically differ in age, weight, height, or body mass index (Table 1). DM1 patients had an average adherence of 98% during the exercise intervention, which consisted of 3 training sessions weekly for 12 weeks on a cycle ergometer (Figure 1B and Table 1). To ensure the safety and assess potential benefits of our exercise protocol, we measured circulating fasting blood glucose (GLUF), creatine kinase (CK), creatinine, bilirubin, alanine transaminase (ALT), and γ-glutamyl transpeptidase (GTT) before and after cycling. Paradoxically, GLUF significantly increased following exercise; however, no differences were observed in other circulating factors that were measured (Supplemental Table 1; supplemental material available online with this article; https://doi.org/10.1172/JCI156125DS1). We next analyzed muscle cross sections for centrally nucleated fibers (CNFs) and other myopathic characteristics. DM1-PRE muscle presented with the expected central nucleation in approximately 27% of fibers compared with approximately 5% in CON. Exercise did not alter the frequency of CNFs (Figure 2, A and B; *P* < 0.05). H&E slides were further subjected to a blinded pathology examination by a neuropathologist for other indicators of muscle damage. No differences in pathology scores were seen between DM1-PRE and DM1 patients after (DM1-POST) exercise (Figure 2C and Supplemental Figure 1). Thus, exercise appears to be safe and well tolerated by DM1 patients, as suggested by blood markers and muscle histopathology examination.

### Exercise modestly improves functional respiration.

Considering respiratory failure is the leading cause of mortality in DM1 (25) and the well-known benefits of aerobic exercise on respiratory function (26, 27), we performed spirometry testing. Forced vital capacity (FVC) and forced expiratory volume (FEV1) were significantly lower in DM1-PRE (~32% and ~36%, respectively) compared with in CON (Table 2 and Supplemental Table 2). No changes were observed in FVC following 12 weeks of exercise (Table 2). Absolute and relative FEV1 values were increased by approximately 5% and approximately 7%, respectively, in response to training, but did not reach statistical significance (unadjusted *P* = 0.12 and 0.067, respectively). To further elucidate the influence of training on FEV1, we performed Pearson’s correlation to evaluate the relationship between baseline FEV1, expressed as a percentage of predicted, and percentage change in FEV1 following exercise. We observed a significant correlation (*r* = –0.62; Supplemental Figure 2) between relative FEV1 values and exercise-induced FEV1 improvements, suggesting that patients with limited respiratory function experience greater benefits.

### Cardiac assessment in DM1 patients.

Subsequently, we examined the influence of exercise on cardiac conduction defects, the second leading cause of death in DM1. At baseline, patient 12 (P12) had a prolonged PR interval, P6 and P9 had an extended QRS complex, and P8 had irregular PR and QRS durations. Following 12 weeks of cycling, P12, P6, and P8 ECG readings remained abnormal, while P9 experienced a minor decrease (~2 ms) in QRS duration. Overall, no significant difference was observed in QRS complex, but PR interval significantly increased, by approximately 8.9 ms, after training (Table 2 and Supplemental Table 2), a commonly seen phenomenon following chronic aerobic exercise in healthy individuals (28, 29). Finally, we assessed heart rate (HR) during a graded exercise stress test. DM1 patients had a significantly lower absolute HR compared with CON at submaximal and maximal exercise intensities, but these differences were no longer evident when normalized to maximal HR (Supplemental Figure 3, A and B). Finally, training did not alter the absolute or relative HR response during acute exercise.

### Cycling improved fitness and function, but not strength.

Whole-body maximal oxygen consumption is strongly associated with overall health and life span. Therefore, we assessed the efficacy of exercise on cardiorespiratory fitness (VO_2peak_). CON had greater (*P* < 0.05) VO_2peak_ (~1.93-fold) and peak power output (W_peak_; 2.69-fold) compared with DM1-PRE (Table 2). Exercise significantly increased relative VO_2peak_ in DM1 patients (~1.32-fold). Concomitantly, W_peak_ increased (*P* < 0.05) by approximately 1.35-fold following training (Table 2 and Supplemental Table 2). Next, we implemented 6-minute walk (6-MWT), timed up-and-go (TUG), and 5× sit-to-stand (5XSTS) tests to assess muscular endurance, mobility, and functional strength, respectively. As anticipated, DM1-PRE had a significantly lower functional capacity compared with CON. Healthy CON outperformed (*P* < 0.05) patients by approximately 1.57-, approximately 1.32-, and approximately 1.34-fold during the 6-MWT, TUG, and 5XSTS tests, respectively (Table 2). After exercise, DM1 patients travelled approximately 47 m further (*P* < 0.05) during 6-MWT (Table 2). Moreover, TUG and 5XSTS significantly improved, by approximately 1.14-fold and approximately 1.21-fold, respectively. We then examined the influence of exercise on maximal strength. Cycling had no effect on maximal knee-extension strength, grip strength, or pinch grip (Table 2 and Supplemental Table 2). Taking these data together, exercise training augmented cardiorespiratory fitness and muscle function without evidence of deleterious histological consequences.

### Cycling increased muscle mass in DM1 patients.

Although resistance exercise is the primary mode of training for muscle growth, some evidence supports the role of aerobic training on skeletal muscle hypertrophy (30). Thus, we investigated the influence of cycling on total lean mass (TLM) in DM1 patients. In line with the muscle-wasting nature of the disease, DM1-PRE had significantly lower TLM compared with CON (Table 2). Exercise enhanced (*P* < 0.05) TLM by an average of approximately 1.6 kg and modestly decreased total body fat percentage, by approximately 2% (Table 2). To confirm that training increased skeletal muscle mass, we employed myosin heavy chain (MHC) staining. Fiber-type distribution analysis revealed a significantly greater proportion of type IIA glycolytic fibers in DM1-PRE compared with CON (Figure 2, D and E). Myofiber cross-sectional area (CSA) increased by approximately 30% (5981 ± 530 μm^2^ to 7925 ± 1060 μm^2^, Figure 2F) independently of fiber type (*P* = 0.052). We observed a non–statistically significant increase in CSA of type I (unadjusted *P* = 0.06) fibers following cycling (Figure 2F). Exercise training notably reduced (*P* < 0.05) the frequency of smaller atrophic type I fibers and increased the frequency of larger hypertrophic fibers (Figure 2G), with no changes seen in type IIA (Figure 2H). Collectively, these results showed that moderate intensity cycling augmented muscle mass, promoted CSA hypertrophy, and improved body composition in DM1 patients.

### Exercise increased MBNL2 levels.

**Figure 3:**
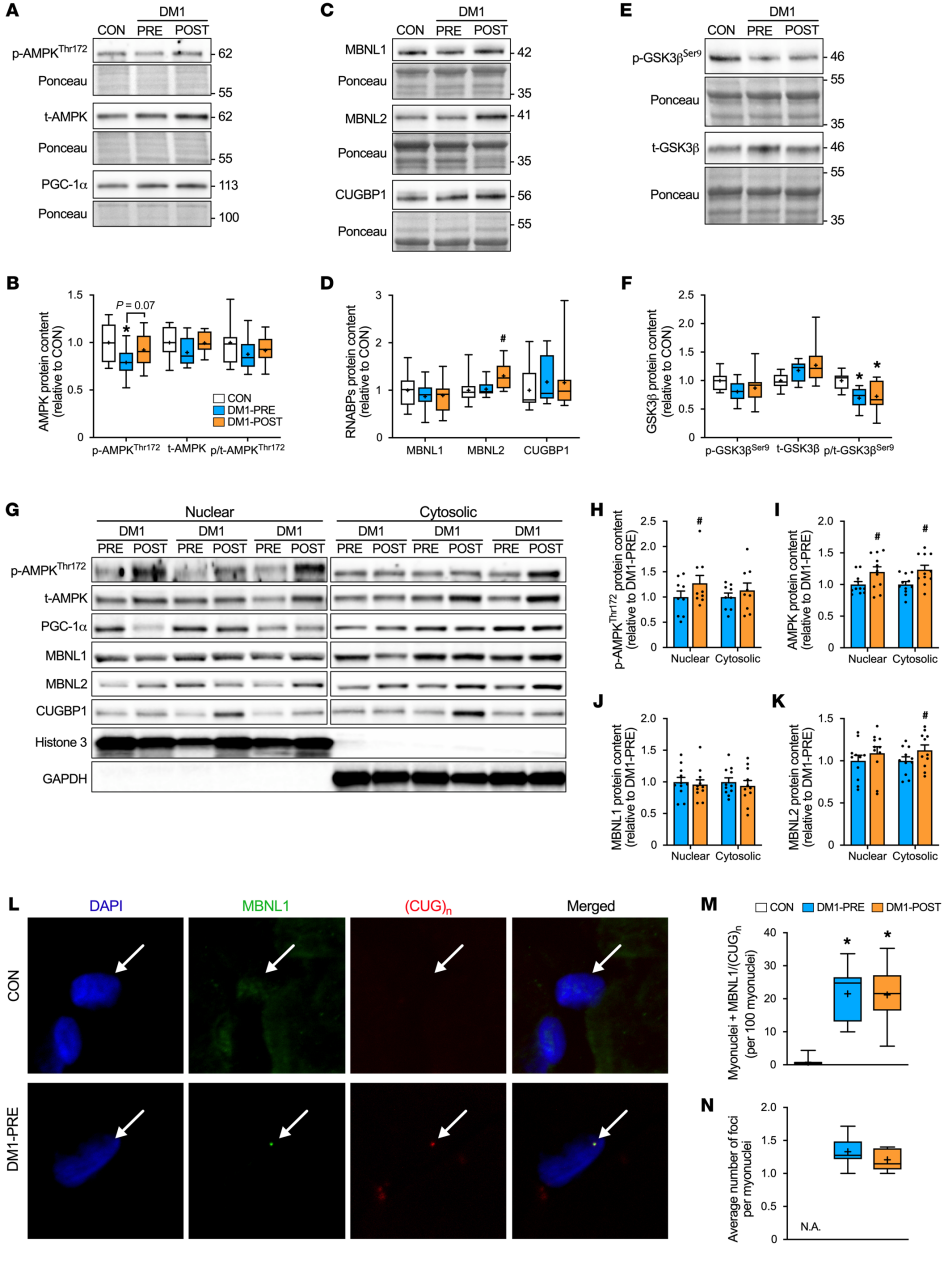
Aerobic exercise modestly increased cytosolic content of MBNL2, but does not alter MBNL1 sequestration. (**A**) Representative Western blot of p-AMPK^Thr172^, t-AMPK, and PGC-1α in the vastus lateralis muscle. (**B**) Graphical summary of p-AMPK^Thr172^, t-AMPK, and PGC-1α expression. (**C**) Representative Western blot of MBNL1, MBNL2, and CUGBP1. (**D**) Graphical summary of MBNL1, MBNL2, and CUGBP1 expression. (**E**) Representative Western blot of p-GSK3β^Ser9^ and t-GSK3β. (**F**) Graphical summary of p-GSK3β^Ser9^, t-GSK3β, and inhibition status (p-GSK3β^Ser9^ relative to t-GSK3β) expression. A typical Ponceau stain displayed below demonstrates sample loading. (**G**) Representative Western blot of p-AMPK^Thr172^, t-AMPK, MBNL1, MBNL2, and CUGBP1 in nuclear and cytosolic fractions from DM1-PRE and DM1-POST. Histone 3 and GAPDH proteins displayed below to indicate nuclear and cytosolic fraction purity. Approximate molecular weights (kDa) shown at right of blots in **A**, **C**, and **E**. (**H–K**) Graphical summary of p-AMPK^Thr172^, t-AMPK, MBNL1, and MBNL2 in nuclear and cytosolic fractions. (**L**) Representative images of combined FISH probing for CUG repeats ([CUG]_n_) and IF staining of MBNL1 along with DAPI to mark myonuclei and merged image. Original magnification, ×60 with a ×10 digital imaging zoom, for final magnification of ×600. (**M**) Summary of the numbers of MBNL1/(CUG)_n_-positive myonuclei as an indicator of MBNL1 sequestration. (**N**) Average number of foci within MBNL1/(CUG)_n_-positive myonuclei. Data are expressed as box and whisker plots with plus signs representing the mean (**B**–**F**, **M**, and **N**) or bar graphs as mean ± SEM (**H**–**K**). *n* = 9–11. *P < 0.05 versus CON, 1-way ANOVA followed by Bonferroni’s correction; ^#^*P* < 0.05 versus DM1-PRE, 2-tailed paired *t* test corrected for multiple comparisons.

Activation and subsequent nuclear migration of AMPK and peroxisome proliferator–activated receptor γ coactivator-1 α (PGC-1α) have been proposed to ameliorate RNA-mediated toxicity in DM1 mice (12–14, 20). Therefore, we assessed the influence of aerobic training on basic DM1 biology. We first measured phosphorylated AMPK (p-AMPK^Thr172^), total AMPK (t-AMPK), and PGC-1α protein content. DM1 patients showed a lower (*P* < 0.05) expression of p-AMPK^Thr172^ compared with CON, but no changes were observed in t-AMPK or PGC-1α protein content (Figure 3, A and B). Exercise training increased p-AMPK^Thr172^ levels in whole muscle lysate (*P* = 0.07) as well as in nuclear fractions (Figure 3, B and H; *P* < 0.05). Similarly, t-AMPK levels increased in nuclear and cytosolic compartments (Figure 3, G and I), but no changes were observed in total, nuclear, or cytosolic PGC-1α (Supplemental Figure 4, A and B). Next, we investigated the expression of several RNABPs implicated in DM1 pathogenesis. At baseline, there were no differences in the protein content of MBNL1, MBNL2, and CUGBP1 between DM1 patients and CON (Figure 3, C and D). Glycogen synthase kinase 3β (GSK3β) is a regulator of CUGBP1 activity through cyclin D3–dependent kinase 4 (31). Therefore, we further examined protein levels of phosphorylated GSK3β (p-GSK3β^Ser9^) and total GSK3β (t-GSK3β) as an indirect marker of CUGBP1 activity. Inhibition status (p-GSK3β^Ser9^/t-GSK3β) of GSK3β was significantly lower in DM1-PRE compared with CON (Figure 3, E and F). Exercise training increased (*P* < 0.05) MBNL2 protein levels, but no changes in MBNL1, CUGBP1, or GSK3β inhibition were observed (Figure 3, C–F). Taken together, these results show that exercise had a modest influence on MBNL2 levels, but did not alter protein expression of other RNABPs.

### Aerobic training alters the subcellular localization of MBNL2, but not MBNL1.

Given the spatial importance of RNABPs on their function, we investigated changes in nuclear and cytosolic MBNL1, MBNL2, and CUGBP1 content. We observed a significant increase in MBNL2 cytosolic content, but exercise did not alter the localization of MBNL1 or CUGBP1 (Figure 3, G and J–K, and Supplemental Figure 4C). Although subcellular fractionation provides some insight into the localization of RNABPs, it fails to assess the degree of MBNL1 sequestration. Thus, we employed FISH for CUG mRNA repeats ([CUG]_n_) repeats in combination with immunofluorescence (IF) to detect MBNL1 protein. As anticipated, DM1-PRE had a greater (*P* < 0.05) degree of MBNL1 sequestration (observed in ~21% of myonuclei) compared with CON (<1%; Figure 3, L and M). Cycling did not alter the proportion of MBNL1/(CUG)_n_–positive myonuclei or the average number of foci per nuclei (Figure 3, L–N). In line with these data, total content of *DMPK* transcripts were not influenced by aerobic training in DM1 patients (Supplemental Figure 4D). Overall, exercise increased cytosolic MBNL2, but did not influence the localization of other splicing mediators or *DMPK-*mediated toxicity.

### DM1 transcriptome profile is significantly different from that of healthy CON.

**Figure 4:**
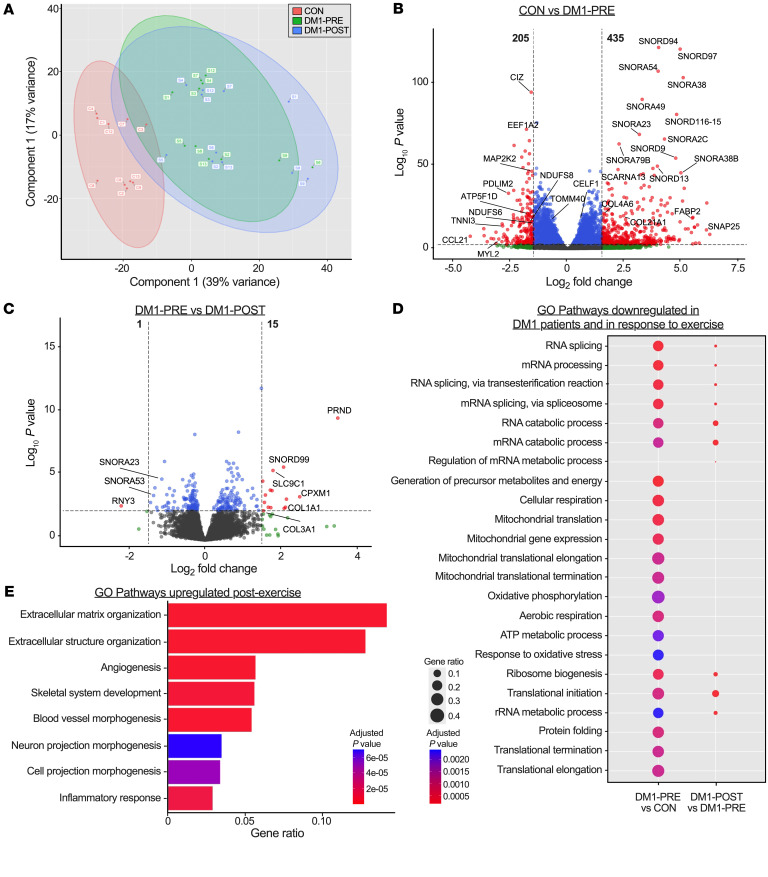
Bulk RNA-Seq reveals a unique transcriptional profile between DM1 patients and healthy CON. (**A**) PCA of bulk RNA-Seq for CON (red), DM1-PRE (green), and DM1-POST (blue). (**B** and **C**) Volcano plots of differential expression analysis between CON versus DM1-PRE (**B**) and DM1-PRE versus DM1-POST (**C**). Significantly different (|log_2_(FC)| > 1.5, *P* < 0.005) genes are indicated with red dots, and nonsignificant genes are indicated in blue (|log_2_(FC)| < 1.5, *P* < 0.005) and green (|log_2_(FC)| > 1.5, *P* > 0.005). (**D**) Bubble plot of downregulated pathways examined through GO pathway analysis. (**E**) GO pathway analysis of biological processes upregulated in DM1 patients in response to exercise training. *n* = 10–11.

To examine the effect of exercise on skeletal muscle transcription in DM1 patients, we performed whole-muscle deep RNA-Seq (~35 million uniquely mappable reads per sample). We first assessed transcriptional differences between DM1-PRE and CON. Principal component analysis (PCA) was performed for all samples and plotted for the first 2 components. CON samples separated from DM1-PRE samples along the first principal component, suggesting a DM1-specific transcriptional profile (Figure 4A). Furthermore, differential expression analysis uncovered 205 genes that were downregulated and 435 genes that were upregulated in DM1-PRE when compared to CON (Figure 4B). Next, we performed Gene Ontology (GO) pathway analysis and identified 3 main biological themes that were downregulated in DM1-PRE: (a) mRNA splicing and metabolism, (b) mitochondrial respiration and translation, and (c) protein translation (Figure 4D and Supplemental Figure 5A). Despite many genes in DM1-PRE being expressed at a higher degree relative to CON, GO pathway analysis did not detect any biological processes that were significantly upregulated (Figure 4B and Supplemental Table 3). Thus, we surveyed the top 50 upregulated genes in DM1-PRE relative to CON, many of which were noncoding RNAs (~80%) belonging to the family of snoRNAs, small nuclear RNAs (snRNAs), and RNY-derived small RNAs (s-RNYs; Supplemental Figure 5B and Supplemental Table 3). Small noncoding RNAs are generally processed out of spliced introns of host genes by exonucleases, and their functions are only partially understood. This includes methylating components of the ribosomal RNA and pseudouridylating enzyme complexes (32). The apparent upregulation of snoRNAs in DM1 occurred in the absence of transcriptional changes in the host gene, suggesting a stabilizing effect on snoRNAs. This is well illustrated with the *SNORD116*/*SNHG14* cluster, which is implicated in Prader-Willi pathogenesis (Supplemental Figure 6), as well as the *SNORA38*/*PRRC2A* and *SNORB38*/*NOL11* loci. Similar changes were noted in snRNA gene levels, which are known to regulate splicing of genes, such as the *RNU5B-1* and *RNU6ATAC* genes (Supplemental Figure 5B). Taken together, our data highlight the transcriptomic differences between DM1 patients and healthy CON subjects primarily due to distinctions in transcripts that regulate mRNA metabolism, oxidative phosphorylation (OXPHOS), and noncoding RNAs involved in ribosomal RNA processing and splicing.

### Chronic training does not alter the basal transcriptional profile in DM1.

Next, we investigated exercise-induced transcriptional changes in DM1 skeletal muscle. DM1-PRE and DM1-POST samples considerably overlapped along the first 2 principal components (Figure 4A), suggesting a minor effect of exercise on the DM1 transcriptome. Furthermore, differential expression analysis revealed 1 downregulated and 18 upregulated genes in response to training (Figure 4C). Genes related to extracellular matrix remodeling, angiogenesis, and inflammation were upregulated in DM1 patients following aerobic training (Figure 4E), which is similar to what has been previously documented in unaffected healthy CON after training (33). Overall, endurance exercise stimulated the expression of genes known to respond to aerobic training, but did not alter any large preexistent transcriptional differences in DM1 patients.

### snoRNA expression is correlated with clinically meaningful measures in DM1 patients.

**Figure 5:**
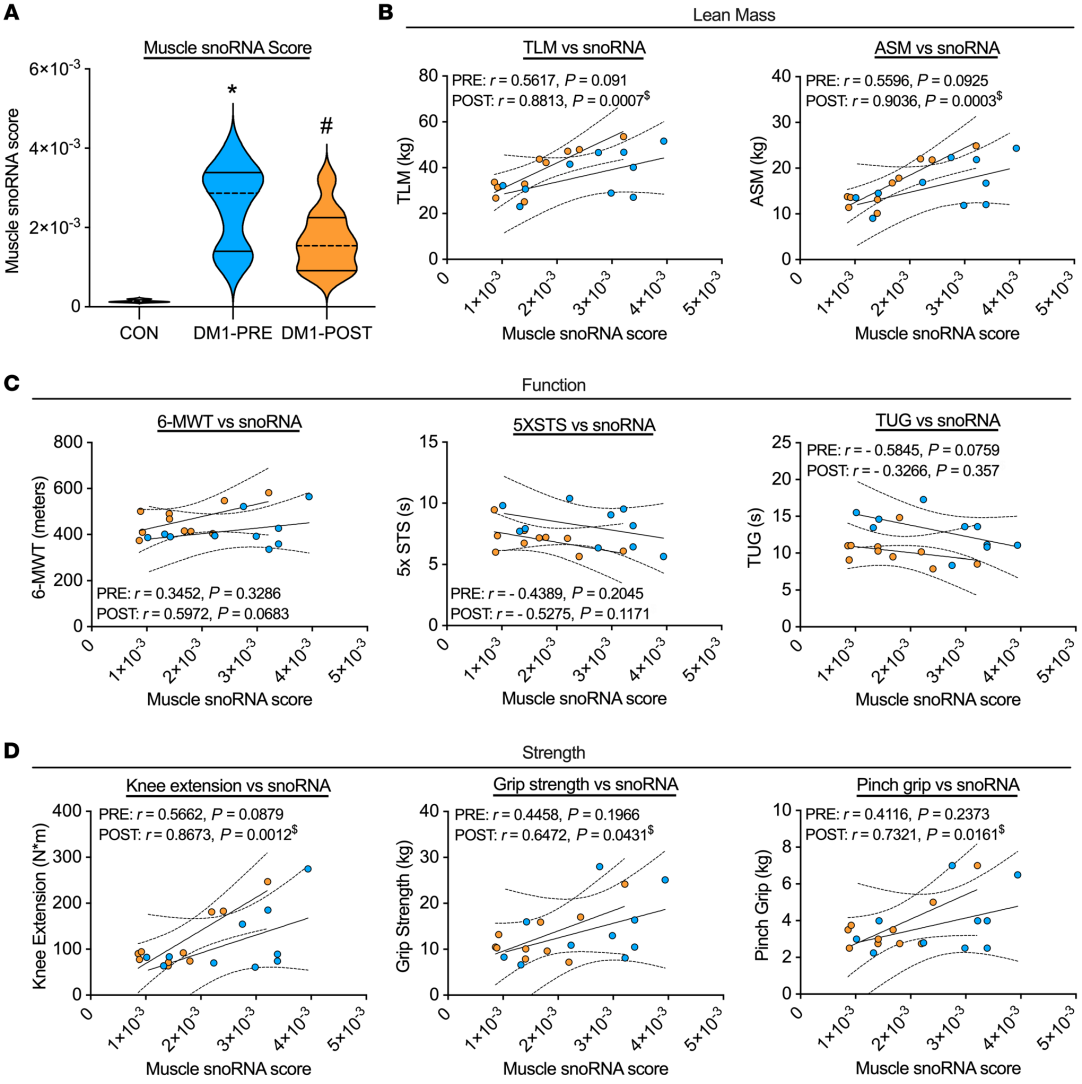
snoRNAs are upregulated in DM1 patients and are correlated with clinical outcomes. (**A**) Violin plot of computed muscle snoRNAs score in CON, DM1-PRE, and DM1-POST. Correlation graphs and values of the muscle snoRNA score against metrics of (**B**) lean mass (TLM and appendicular skeletal muscle [ASM]), (**C**) function (6-MWT, 5XSTS, and TUG tests), and (**D**) strength (maximal knee extension, grip strength, and pinch grip). *n* = 10. *P < 0.05 versus CON, 1-way ANOVA followed by Bonferroni’s correction; ^#^*P* < 0.05 versus DM1-PRE, 2-tailed paired *t* test corrected for multiple comparisons; ^$^*P* < 0.05, linear regression analysis.

To determine the physiological relevance of the robust increase in snoRNAs within DM1 patients, we developed a muscle snoRNA score as a proxy for overall content (Supplemental Table 4) and correlated it with lean mass, strength, and function. In line with bulk RNA-Seq data, our computed muscle snoRNA score was approximately 18-fold greater in DM1-PRE relative to CON (Figure 5A). Remarkably, muscle snoRNA scores positively correlated with variables related to lean mass, muscle function, and strength, with the strongest correlations being TLM, appendicular skeletal muscle mass, and knee extension strength (Figure 5, B–D). Following exercise training, we observed a significant reduction in the levels of several snoRNAs (Figure 5A and Supplemental Figure 6). Furthermore, the relationship between muscle snoRNA score and metrics of muscle mass, strength, and function were strengthened in DM1 patients following the exercise intervention. Our data suggests that snoRNA expression may be physiologically relevant within DM1 biology.

### Exercise training does not correct alternative splicing in DM1.

**Figure 6:**
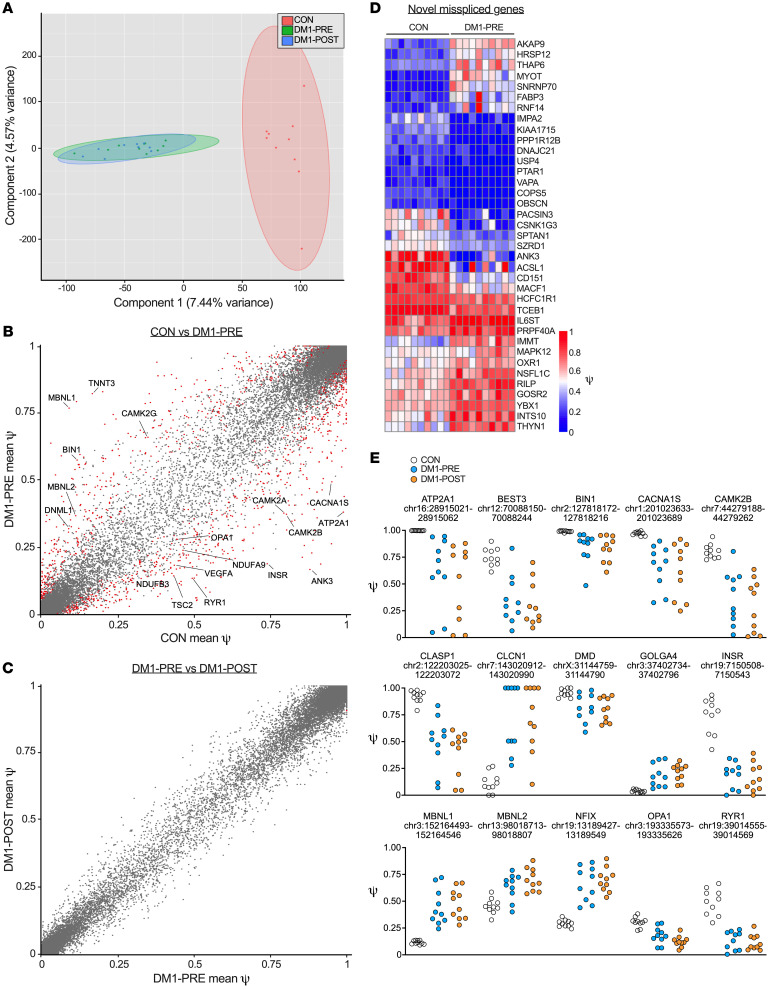
Twelve weeks of cycle ergometry does not influence missplicing in skeletal muscle of DM1 patients. (**A**) PCA of whole-genome exon splicing data examining differences in genome-wide alternative splicing events for CON (red), DM1-PRE (green), and DM1-POST (blue). (**B** and **C**) Scatterplots of mean PSI (or ψ) for (**B**) healthy CON versus DM1 samples before exercise and (**C**) DM1 patients before (DM1-PRE) and after (DM1-POST) exercise for all exons measured; 1581 missplicing events were detected as significantly different (|ψ| > 5%, FDR < 5%, *P* < 0.0002). Red dots represent significantly different alternatively spliced events, and gray dots represent nonstatistically different event. (**D**) Heatmap showing ψ values of novel alternatively spliced events in skeletal muscle of DM1 patients relative to healthy CON. (**E**) Scatterplots of individual ψ values for canonical missplicing events in DM1 biology. *n* = 10.

We next sought to investigate changes in misspliced events in DM1 following cycling. We used rMATS to analyze genome-wide pre-mRNA alternative splicing by calculating the percentage spliced in (PSI or ψ) for all samples. We performed PCA on all alternatively spliced events and observed a strong separation between DM1-PRE and CON along the first principal component (Figure 6A). We identified a total of 1581 uniquely spliced events between DM1-PRE and CON (Figure 6B and Supplemental Table 5). Interestingly, we discovered 37 misspliced genes that, to our knowledge, have not been previously reported (Figure 6D and Supplemental Table 6). We further examined any splicing alterations brought about by exercise through a pair-wise comparison between DM1-PRE and DM1-POST samples. Contrary to previous studies in DM1 mice (13, 14), exercise training failed to reverse canonically misspliced events (Figure 6, C and E, and Supplemental Figure 7). Similarly, no changes were observed in the exclusion of *MBNL1* exon 5 (Figure 6E), a negative regulator of MBNL1-splicing activity and a major contributor to the missplicing in DM1. Collectively, our transcriptome-wide alternative splicing assessment was able to detect both previously discovered and what we believe to be novel misspliced events. Additionally, the robust improvements in fitness and function seen following endurance training in DM1 patients occurred independently of adaptations in DM1-associated spliceopathy.

### Exercise training ameliorates mitochondrial deficiency in DM1.

**Figure 7:**
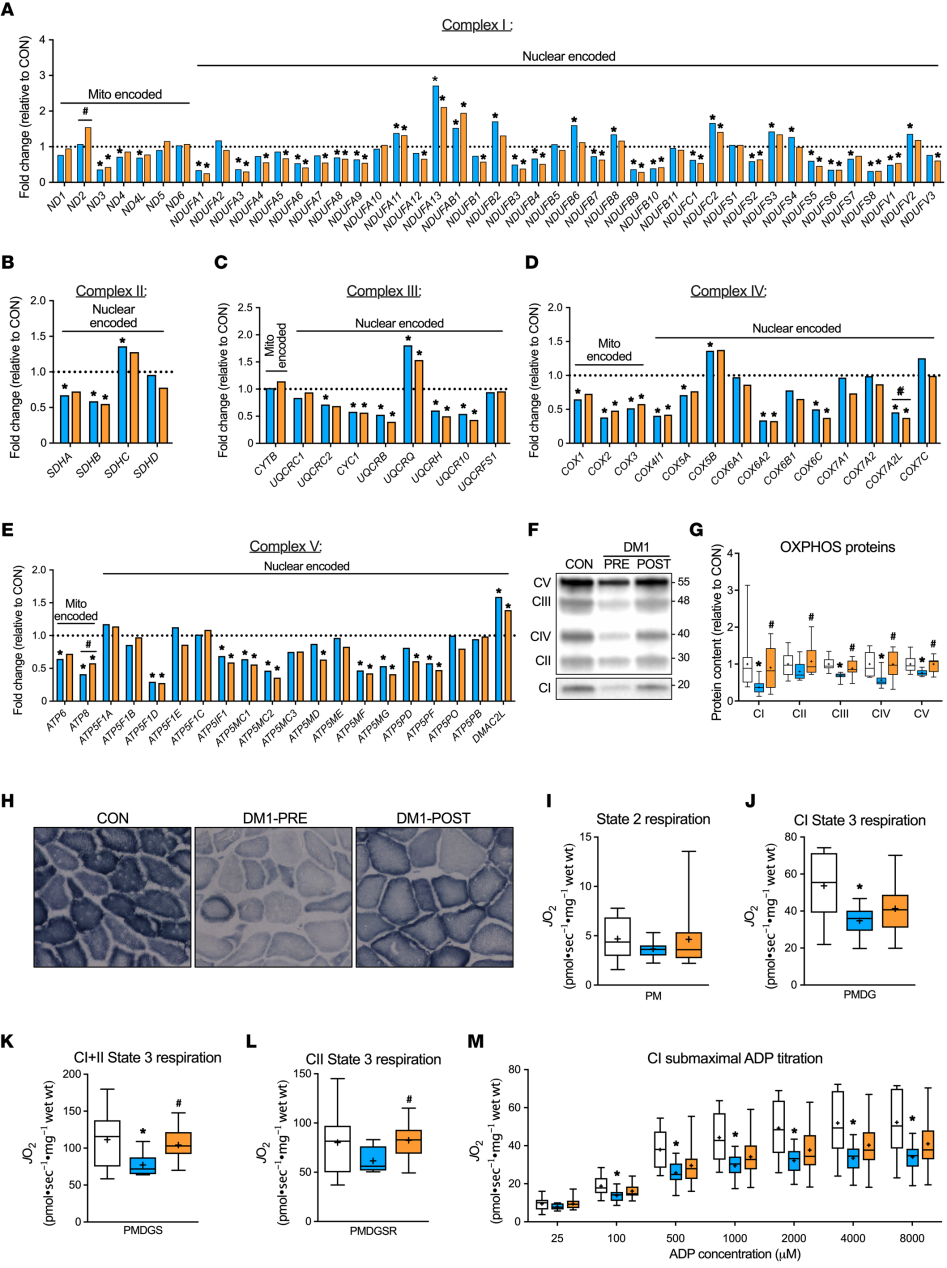
Exercise training ameliorates mitochondrial deficiency in DM1 patients. Gene expression of all subunits of complex I (**A**), complex II (**B**), complex III (**C**), complex IV (**D**), and complex V (**E**) of the mitochondrial electron transport chain expressed as fold change relative to CON and grouped into mitochondrial (left) and nuclear encoded genes (right). (**F**) Representative Western blot of mitochondrial protein complexes (CI–CV). Full blot was overexpressed and cropped for better visualization of CI. A typical Ponceau stain displayed below demonstrates sample loading. Approximate molecular weights (kDa) shown at right of blots. (**G**) Graphical representation of CI–CV protein expression. (**H**) Representative images of succinate dehydrogenase staining. Original magnification, ×20. (**I**) State 2 complex I (CI) respiration in the presence of pyruvate plus malate (PM). (**J**) State 3 CI maximal respiration in the presence of PM plus ADP plus glutamate (PMDG). (**K**) State 3 complex I+II (CI+II) maximal respiration in the presence of PMDG plus succinate (PMDGS). (**L**) State 3 complex II (CII) maximal respiration in the presence of PMDGS plus rotenone (PMDGSR). (**M**) Submaximal ADP titration (25, 100, 500, 1000, 2000, 4000, and 8000 μM) curve with PM. Two samples from CON were of poor quality and therefore excluded from the respiration analysis. All respiration experiments were performed in duplicate and averaged for each participant. Data are expressed with bar graphs as mean (**A**–**E**) or as box and whisker plots with plus signs representing the mean (**G** and **I–M)**. *n* = 9–11. *P < 0.05 versus CON, 1-way ANOVA followed by Bonferroni’s correction; ^#^*P* < 0.05 versus DM1-PRE, 2-tailed paired *t* test corrected for multiple comparisons.

Since exercise substantially improved clinical and functional outcomes in patients independently of alteration to the classic DM1 mRNA toxicity, we investigated posttranscriptional factors related to mitochondrial content and function, as they were the second most downregulated biological process in DM1 patients. In line with GO pathway analysis, the expression of mRNA transcripts coding for OXPHOS proteins were significantly downregulated in DM1-PRE (Figure 7, A–E). The AMPK/PGC-1α signaling axis is critical for maintaining mitochondrial content and quality (34). In agreement with lower levels of p-AMPK^Thr172^, OXPHOS proteins were significantly lower in DM1-PRE. Specifically, complex I, III, IV, and V proteins were blunted (*P* < 0.05) by 0.37-, 0.68-, 0.56-, and 0.77-fold, respectively, in DM1-PRE relative to CON (Figure 7, F and G). Lower protein abundance was accompanied by reduced (*P* < 0.05) ADP-stimulated submaximal and maximal respiration of complex I (Figure 7, J and M) as well as maximal complex I+II–specific state 3 respiration (~31 %) in DM1 patients (Figure 7K). No differences were observed in complex I state 2 or complex II state 3 respiration (Figure 7, I and L)

Aerobic training increased (*P* < 0.05) protein content of mitochondrial complexes to levels comparable to those of CON (Figure 7G). Furthermore, qualitative analysis revealed an increase in succinate dehydrogenase (SDH) staining intensity in DM1-POST compared with DM1-PRE (Figure 7H). Oxygen consumption rates (OCR) of complex I+II and II state 3 respiration were enhanced (*P* < 0.05) in DM1-POST (Figure 7, K and L). Complex I submaximal respiration rates in DM1 patients after exercise were no longer statistically different from those of CON (Figure 7M). To obtain predicted values of ADP sensitivity, we employed Michaelis-Menten analysis to calculate an estimated apparent Km. There were no significant differences in apparent Km among all groups (Supplemental Figure 8). Overall, DM1 patients presented with reduced mitochondrial transcripts, protein abundance, and function, which were mitigated with exercise training only at a posttranscriptional level.

### Exercise alters expression of proteins important for mitochondrial dynamics.

**Figure 8:**
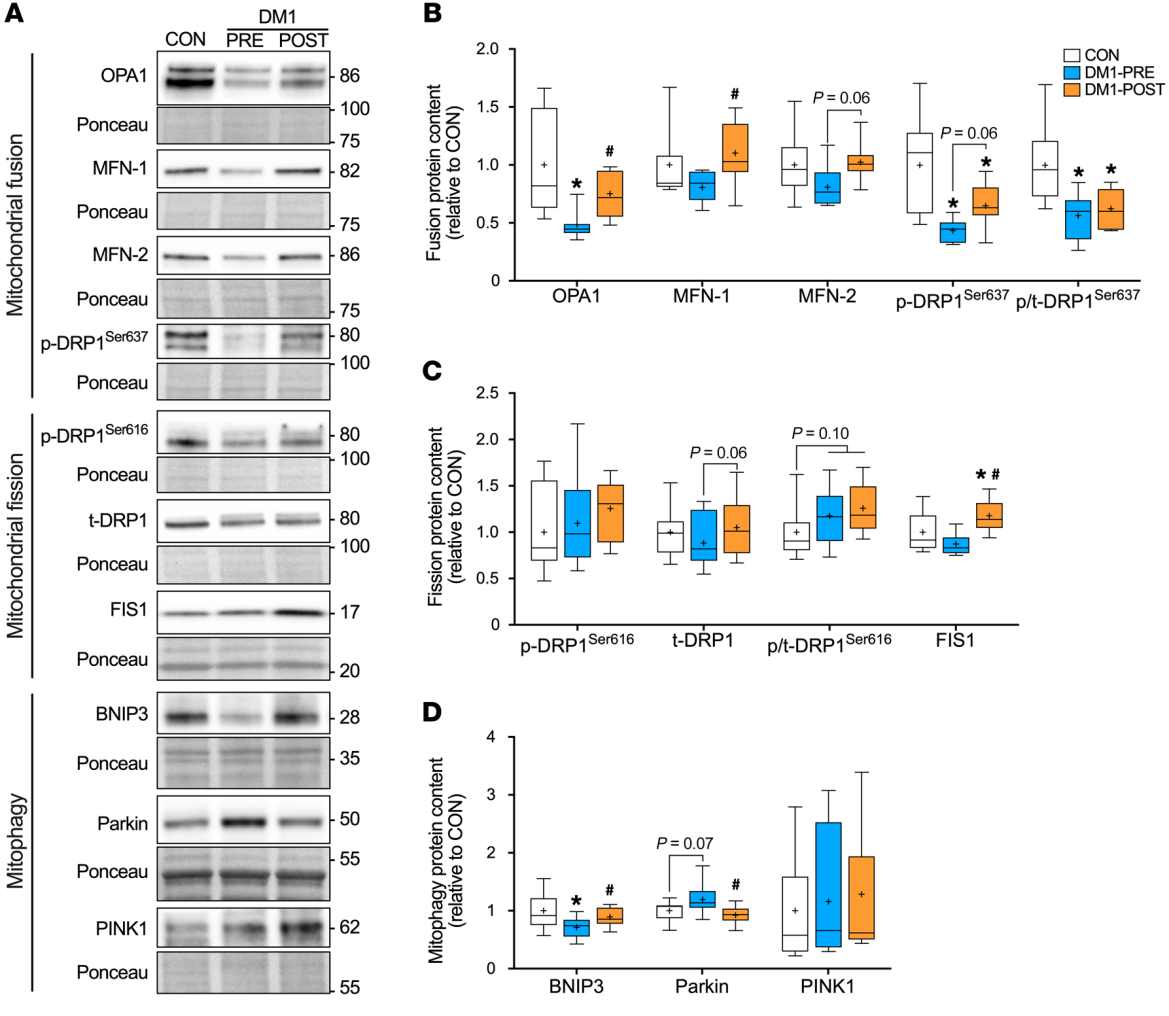
Aerobic exercise augments proteins that regulate mitochondrial plasticity in skeletal muscle of DM1 patients. (**A**) Representative Western blot of mitochondrial fusion-related proteins (OPA1, MFN-1, MFN-2, and p-DRP1^Ser637^), fission-related proteins (p-DRP1^Ser616^, FIS1, and t-DRP1), and mitophagy proteins (BNIP3, Parkin, and PTEN-induced kinase [PINK1]). A typical Ponceau stain displayed below demonstrates sample loading. Approximate molecular weights (kDa) shown at right of blots. (**B**) Graphical summary of OPA1, MFN-1, MFN-2, p-DRP1^Ser637^, and inhibition status for DRP1 (p-DRP1^Ser637^ relative to t-DRP1) expression. (**C**) Graphical summary of p-DRP1^Ser616^, t-DRP1, and FIS1 expression. (**D**) Graphical summary of BNIP3, Parkin, and PINK1 expression. All data are expressed as box and whisker plots with plus signs representing the mean. *n* = 11. *P < 0.05 versus CON, 1-way ANOVA followed by Bonferroni’s correction; ^#^*P* < 0.05 versus DM1-PRE, 2-tailed paired *t* test corrected for multiple comparison.

Mitochondrial morphology is a key determinant of organelle function (35). We examined expression of proteins essential for mitochondrial plasticity. At baseline, DM1 patients showed lower optic atrophy protein 1 (OPA1) (*P* < 0.05) and mitofusin-2 (MFN-2) (unadjusted *P* = 0.062) protein levels. Additionally, the inhibitory phosphorylation site of dynamin-related protein 1 (p-DRP1^Ser637^) was approximately 57% lower (*P* < 0.05) in DM1-PRE relative to CON (Figure 8, A and B). MFN-1 protein levels were similar at baseline between groups, as were fission-related proteins total DRP1 (t-DRP1) and mitochondrial fission 1 (FIS1) (Figure 8, B and C). We observed a trending increase in DRP1 activation (p-DRP1^Ser616^/t-DRP1; *P* = 0.10) in DM1-PRE compared with CON. Finally, mitophagy-related protein BCL2 interacting protein 3 (BNIP3) was significantly lower in DM1-PRE, but its downstream target, Parkin, was more abundant (*P* = 0.07, Figure 8D). In response to aerobic training, OPA1, MFN-1, FIS1, and BNIP3 levels significantly increased (Figure 8, A–D). Similarly, MFN-2, p-DRP1^Ser637^, and t-DRP1 increased following exercise, but increases failed to reach statistical significance (*P* = 0.06, *P* = 0.08, and *P* = 0.06, respectively; Figure 8, A–C). In contrast, Parkin protein levels were normalized (*P* < 0.05) to CON levels (Figure 8D). Collectively, these data suggest that there was an imbalance among fusion-, fission-, and mitophagy-related proteins in DM1 patients that was partially restored following 12 weeks of aerobic training.

## Discussion

DM1 is a multifaceted life-limiting disorder that can severely affect the health of individuals affected. With no current cure, there is a critical need for an intervention to alleviate DM1 progression and improve patients’ quality of life. Our study demonstrates the efficacy of moderate intensity cycling in eliciting several clinical and physiological benefits in DM1. Mechanistically, these adaptations were underpinned by augmented mitochondrial content and function, but no changes to the core spliceopathy observed in skeletal muscle of patients with DM1. Additionally, we provide the first evidence, to our knowledge, to support the dramatic changes in snoRNAs as well as other noncoding RNAs in DM1 biology. Collectively, the cycling protocol described herein is a safe and effective mode of exercise that should be included in clinical practice to mitigate skeletal muscle wasting and improve overall health in DM1.

The progressive and relentless nature of DM1 results in a substantial burden for patients in performing activities of daily living and severely affects their health (36). As a result, many repurposed small molecules are currently being examined in different preclinical and clinical stages. Metformin (an AMPK activator), tideglusib (a GSK3β inhibitor), and mexiletine (an anti-myotonia drug) are among the leading candidates with potential for approval in DM1 (15, 37–39). However, to date, there is a lack of treatment or intervention that safely elicits clinically meaningful outcomes for DM1 patients. In this study, we propose that exercise may be a promising therapy for mitigating muscle and body compositional aspects of DM1 progression and provide strong evidence for clinical adaptations. Twelve weeks of cycling dramatically increased cardiorespiratory fitness, muscular endurance, and mobility and augmented TLM mass and myofiber CSA, in turn, likely improving quality of health and decreasing disease burdens on daily tasks. Additionally, our data suggest that cycling improved metrics of functional respiration in those most susceptible to respiratory failure (Table 2 and Supplemental Figure 2). This is crucial given the progressive decline in lung vital and functional capacity over time in DM1 patients (40). In contrast, clinical trials investigating the efficacy of metformin reported inferior benefits in 6-MWT (+33 m versus +47 m in the current trial), while a mexiletine trial failed to show any improvements in muscular or lung function (15, 38). In line with the present study, a 12-week resistance training program remarkably improved muscle function and strength in 11 males with DM1 (41). Therefore, exercise training appears to provide superior clinical benefits compared with mexiletine and metformin.

Trials examining the safety of resistance, aerobic, or mixed training have all concluded that exercise has no detrimental effects on DM1 patients (see ref. 21 for summary). Similarly, our data support the safety of cycling, as indicated by the lack of changes in DM1-associated myopathy (Figure 2, A–C, and Supplemental Figure 1) as well as circulating factors that would be considered deleterious (Supplemental Table 1). No adverse events were reported during this 12-week trial, while several drug-related adverse events were documented following metformin and mexiletine administration, most of which were related to gastrointestinal issues (15, 38). Finally, it was important to monitor ECG readings before and after exercise, as it was not previously examined. The approximately 9 ms increase in PR interval following exercise (Table 2 and Supplemental Table 2) was not unexpected, as first-degree atrioventricular blocks are commonly seen in healthy aerobically trained individuals (28, 29, 42). Furthermore, a recent clinical trial in DM1 patients reported similar increases in the PR interval following a 6-month follow-up period in both the placebo and mexiletine groups (38), indicating a partial effect of aging per se. Thus, our evidence conforms with previous literature and reiterates that exercise is safe in DM1 patients.

Understanding the underlying molecular mechanisms that lead to exercise-mediated physiological benefits is crucial, as this would provide further insight for discovery of pharmaceutical or other therapies for DM1. AMPK has been extensively studied for its therapeutic potential in the preclinical and clinical context of NMDs (12). For DM1, Savkur et al. (43) were the first to suggest that AMPK activation, through metformin treatment, corrected the metabolic dysfunction observed in DM1 cells. Several others followed to study the mechanism through which the heterotrimeric kinase can ameliorate DM1 pathology. AICAR- and exercise-induced activation of AMPK in DM1 mice resulted in functional improvements and reduced myotonia, which were largely driven by a reduction in CUG foci, liberating MBNL1 and correcting several missplicing events (13, 14). Although the mechanism is poorly understood, it is believed that once stimulated, AMPK will augment PGC-1α activity, resulting in its translocation to the nucleus and modulating the function of important splicing factors and RNA polymerase II (44, 45). Alternatively, nuclear accumulation of AMPK can increase its binding to heterogeneous nuclear ribonucleoprotein H (hnRNP H), thereby reducing its activity (46) and destabilizing the CUG hairpin loops, in turn freeing MBNL1 (47). Here, we demonstrate that exercise training increased nuclear content of AMPK and its active form, p-AMPK^Thr172^ (Figure 3, G–I). However, aside from a modest increase in cytosolic MBNL2 protein (Figure 3K), we found no changes in *DMPK*-driven toxicity. In contrast to investigations in DM1 mice, exercise-mediated AMPK activation in DM1 patients failed to increase cytosolic MBNL1 content, reduce MBNL1/CUG-positive myonuclei, or alter the DM1-associated transcriptome (Figure 3 and Figure 4C).

Upregulation and hyperactivation of CUGBP1 have been suggested to further exacerbate DM1 spliceopathy. In the present study, we provide strong evidence of missplicing in skeletal muscle of DM1 patients without any observed increases in CUGBP1 abundance. These results are consistent with previously published data of 18 DM1 patient biopsy samples by Cardani et al. (48), which also demonstrated no significant difference in CUGBP1 levels between healthy and DM1 participants. Nevertheless, it is important to note that overall abundance is not indicative of CUGBP1 activity. Direct assessment of CUGBP1 activity has several methodological challenges, specifically in human skeletal muscle, and requires further investigations. We used GSK-3β inhibition to provide indirect insight of CUGBP1 activity and found that GSK-3β was inhibited to a lesser extent in DM1 skeletal muscle, potentially suggesting augmented CUGBP1 activity that was unaffected by exercise (Figure 3 and Figure 6). These results ultimately indicate that robust increases in CUGBP1 protein levels are not present within proximal skeletal muscle biopsies of DM1 patients and that MBNL1/2 sequestration is likely the major contributor to the abnormal splicing in DM1. Furthermore, the lack of changes to major pathological aspects of the disease suggests an alternative mechanism through which aerobic exercise could mitigate DM1 pathophysiology in patients.

Mitochondrial content, quality, and function are crucial for skeletal muscle maintenance and remodeling. Investigations of mitochondrial health in DM1 biology are scarce, with several studies lacking direct measures of OXPHOS in patients. Several studies conducted prior to the discovery of the DM1 genetic mutation reported the presence of ragged red fibers and cytochrome *c* oxidase negative fibers in DM patients (49–51). More recently, skeletal muscle of DM1 patients displayed aberrant phosphocreatine dynamics, suggestive of poor mitochondrial function (52). Similarly, patient-derived fibroblasts experience reduced OCR and abnormal mitochondrial plasticity (53), yet direct exploration of mitochondrial health in skeletal muscle of genetically confirmed DM1 patients is lacking. Herein, we propose that, second to RNA toxicity, mitochondrial dysfunction is a key contributor to the DM1 phenotype (Figure 4D). Our data demonstrated that mitochondrial transcription, protein abundance, and respiration are severely downregulated in skeletal muscle of DM1 patients (Figure 7). Furthermore, the expression profile of proteins regulating mitochondrial dynamics suggested a more fragmented and simplified morphology, as indicated by significantly lower levels of fusion-related proteins (Figure 8). OPA1 protein tightly regulates inner mitochondrial membrane fusion, cristae complexity, and assembly of mitochondrial super complexes under metabolic stress (35). Thus, one could speculate that the severe blunting of OPA1 protein seen in DM1 skeletal muscle further hinders mitochondrial function. Consequently, mitochondrial abnormalities in DM1 skeletal muscle negatively affect aerobic capacity in patients, as evidenced by poor fitness, and are likely contributors to atrophic phenotype.

An acute bout of aerobic exercise can rapidly phosphorylate and activate AMPK and its downstream substrate PGC-1α, leading to transient increases in nuclear genes encoding for mitochondrial proteins as well as important genes that regulate mitochondrial quality control (16). Accordingly, chronic exercise evidently enhances mitochondrial health. In the present study, exercise training considerably increased OCR of complex II and complex I+II and normalized protein content of all mitochondrial complexes to healthy CON levels (Figure 7). Additionally, cycling restored content of several fusion, fission, and mitophagy regulatory proteins (Figure 8). Therefore, our data suggest that improved mitochondrial health and plasticity in DM1 patients following the 12-week intervention is likely the underlying mechanism responsible for exercise-induced clinical and physiological benefits observed and a promising therapeutic avenue for DM1.

Finally, our exploratory RNA-Seq analysis highlighted significant upregulation of snoRNAs and other noncoding RNAs (Supplemental Figure 5B). This is the first paper to our knowledge to detect increased expression of several noncoding RNA families in DM1 skeletal muscle, and thus their role remains unknown. Nonetheless, we aimed to broadly understand whether increased snoRNAs were pathological in nature or a compensatory mechanism to mitigate DM1 progression. Our computed muscle snoRNA score confirmed that snoRNAs are heavily expressed in DM1 skeletal muscle compared with healthy CON and are positively correlated with metrics of skeletal muscle mass, strength, and function (Figure 5). These data suggest that upregulation of snoRNAs may be beneficial within DM1 pathology, as those with greatest expression of snoRNAs had greater muscle mass, strength, and function, demonstrating a potential for snoRNAs to be utilized as biomarkers for DM1 disease progression and severity. Similarly, others have recently highlighted specific microRNAs, another family of small noncoding RNAs, as alternative biomarkers in serum samples of DM1 mice and patients as an indicator of disease progression (54). Interestingly, following 12 weeks of cycling, the expression of several snoRNAs was significantly blunted and its correlation with metrics of muscle health was increased (Figure 5). The role of snoRNAs within the context of exercise physiology is a relatively novel field of study, with some literature suggesting that levels of circulating snoRNAs and other noncoding RNAs may be linked to exercise status (i.e., sedentary versus trained individuals), but to our knowledge, the functions of snoRNA within skeletal muscle biology have yet to be elucidated (55, 56).

A recent review by Roussel and colleagues summarized the limited body of literature on aerobic, strength, or mixed exercise training in DM1 patients and highlighted the lack of trials exploring functional, clinical, and mechanistic efficacy (21). Roussel et al. concluded that the evidence of aerobic exercise training in DM1 is equivocal due to the small sample size and the heterogeneity of exercise modalities, intensities, and outcomes employed in these studies (21). The findings in the current study provide confirmation for the potential of cycling to mitigate/delay aspects of DM1 progression. However, this study is not without limitations. Although we aimed to account for differences in physical activity between participants (<2 hours of structured physical activity per week), the lack of baseline accelerometry data is a drawback of the current study. Whether these dramatic exercise-induced changes are due to deconditioning or whether exercise modifies the trajectory of disease progression remains unknown. Our laboratory has previously shown that DM1 patients who are regularly active outperformed those who were sedentary in numerous strength outcomes, which perhaps provides the most compelling evidence for long-term benefits of exercise in this population (19). Another limitation to this study is the single center design, which limits our sample population, a common challenge in several DM1 exercise studies (24, 41). This may have in fact hindered our ability to detect changes in response to cycling with some of the molecular measures implemented. Future studies should aim to incorporate a greater number of patients by taking a multicenter approach.

In summary, we demonstrate that 12 weeks of moderate intensity cycling can induce substantial clinical, respiratory, physical, and metabolic benefits in previously sedentary DM1 patients. Exercise-induced improvements are largely due to improved mitochondrial quality and content and not due to changes in DM1-associated RNA toxicity or spliceopathy, as previously described in preclinical models. Finally, we provide the first link, to our knowledge, to implicating snoRNA expression levels on metrics of DM1 disease severity.

## Methods

### Participants.

All DM1 patients at the Neuromuscular Clinic at McMaster University Medical Center were considered for this trial. A detailed schematic of the recruitment process can be found in Figure 1A. A total of 13 DM1 patients were interested and were recruited to participate in this trial. P10 and P11 dropped out after week 2 of the trial for personal reasons unrelated to the study. Therefore, a total of 11 participants were included in the final analysis. Leukocyte CTG repeat length was measured for all patients at time of diagnosis and was not measured during this trial. In parallel, 11 inactive age- and sex-matched healthy CON were recruited from the community for reference values. All study participants performed less than 2 hours of structured aerobic activity per week and were asked to refrain from performing any other exercise during the study. Other exclusion criteria included smoking, obesity (BMI >34.9 kg/m^2^), diabetes, cardiovascular or respiratory disorders (other than a mild restrictive ventilatory defect), other genetic disorders, active musculoskeletal injuries, or any other health complications that would preclude them from performing any exercise. Participants enrolled in this trial were not on any medications.

### Study design.

All participants completed 3 baseline visits to assess preexercise measures. Visit 1 included anthropometric measures, body composition, electrocardiogram (ECG), and a VO_2peak_ test. After 48 hours, participants completed visit 2, which consisted of functional, strength and spirometry testing. Finally, participants were asked to refrain from exercise for a minimum of 48 hours and report to our laboratory fasted for visit 3 to collect a blood sample and skeletal muscle biopsy from the vastus lateralis. Only the DM1 group completed 12 weeks of cycling and follow-up testing. Postexercise testing was done the week immediately after the last exercise sessions and consisted of tests from the same 3 visits completed at baseline.

### Training intervention.

Exercise protocol consisted of 3 training sessions per week for a 12-week period on an electronically braked cycle ergometer (Lode). Each training session began with a 3-minute warm-up at 25 watts (W), followed by 30 minutes at 65% of max workload determined during VO_2peak_ test (W_peak_), and ended with a 2-minute cooldown at 25 W. Training intensity progressively increased to 35 minutes at 75%W_peak._ All training sessions were completed in our laboratory and supervised at all times.

### Spirometry and ECG.

Participants performed a series of 3 strong inhalations, followed by a strong exhalation in a seated position to measure FVC and FEV1 (Vyaire Medical). FVC and FEV1 values are expressed as absolute and as a percentage of predicted values. DM1 patients underwent a standard 12-lead ECG (GE Healthcare) before and after performing a VO_2peak_ test to detect arrhythmias. An electrophysiology cardiologist was consulted if any patients had a PR interval greater than 225 ms or QRS complex greater than 125 ms prior to performing any strenuous exercise.

### VO_2__peak_ test.

Peak oxygen uptake was measured using a metabolic cart with a gas collection system (Moxus Modular Metabolic System, AEI Technology) during an incremental cycle ergometer test. The test began with a 2-minute warm-up at 30W, after which the power was progressively increased by 15W and 30W for DM1 and CON participants, respectively, every minute until participants reached volitional exhaustion or a cadence below 50 rpm. HR was continuously monitored throughout the test using a HR monitor (Polar A3). VO_2peak_ was defined as the highest oxygen consumption recorded over a 15-second period, and maximal workload was the highest power output reached during the test.

### Body composition.

A Dual-Energy X-ray Absorptiometry (DEXA) (GE Lunar Prodigy) scan was used to measure fat mass, TLM, and body composition before and after exercise intervention.

### Functional testing.

A total of 3 tests were used to assess functional capacity: 6-MWT, 5XSTS, and TUG tests. The 6MWT score is an indicator of muscular endurance (57). Participants were given standardized instructions, and tests were done in a flat, 15 m hallway. The 5XSTS test was used as a measure of functional strength of the lower limbs, and the TUG test was implemented to assess functional mobility and agility. Both tests were completed as previously described (58, 59).

### Strength testing.

Participants completed isometric unilateral maximal knee extension on a dynamometer (System 3, Biodex Medical Systems) at a 90° angle on the right leg as previously in our laboratory (19). A hand dynamometer was used to assess maximal grip strength (Jamar, Sammons) and lateral/key pinch grip (B&L Engineering) for both the right and left hands. Participants were instructed to maximally contract for 3 seconds with 30 seconds of rest between trials. Measurements were done in triplicate on each hand, and highest force achieved was used.

### Blood analysis.

Blood collection was performed during visits 3 and 6 (for DM1 patients only) under fasted conditions. Heparinized blood was collected in a fasted state and plasma immediately collected following centrifugation. Samples were then sent to the CORE facility at McMaster University Medical Center (CLIA-certified laboratory) for analysis of GLUF, CK, creatinine, bilirubin, ALT, and GTT levels.

### Muscle biopsy.

A resting muscle biopsy was obtained from the mid-section of the vastus lateralis during the last visit of baseline and follow-up testing using a suction-modified Bergström needle, as previously described (60). The biopsy leg was randomized between participants, but before and after biopsies were taken from the same leg with more than 1.5 cm between incisions. Approximately 20 mg of muscle was embedded in OCT medium for histological/IF experiments, and the remaining samples were frozen in liquid nitrogen and stored in a –80° freezer until further analyses.

### Histochemical staining.

OCT-embedded samples were sectioned into 10 μm cross sections on a cryostat (Leica Biosystems) and stained for H&E. Slides were then dehydrated with successive washes of ethanol and further dried with xylene, then mounted with Permount (Thermo Fisher Scientific). Images were captured using the Nikon 90*i* eclipse upright microscope (Nikon Instruments). CNFs were analyzed using ImageJ 2.0 software (NIH) and were defined as a muscle fiber with a minimum of 1 myonuclei not in contact with the periphery. An average of 250 myofibers were analyzed per sample.

### IF staining and analysis.

MHC staining was performed as previously described by our laboratory (61). Antibody parameters can be found in Supplemental Table 6. Fiber type analysis and CSA were done using Nikon NIS Elements AR 4.40 software (Nikon Instruments). The entire muscle sample was analyzed for fiber-type distribution analysis, and approximately 60% of fibers were used to obtain accurate measures for CSA (62). An average of 83, 92, and 38 fibers were circled for type I, type IIA, and type IIX, respectively.

### Protein extraction and Western blotting.

Approximately 20 mg of frozen muscle was powdered using a cell crusher (Cellcrusher), then placed in RIPA buffer (Thermo Fisher Scientific) with Halt protease and phosphatase inhibitor cocktail (Thermo Fisher Scientific). Samples were then sonicated (Qsonica) and incubated at 4°C for 30 minutes. Finally, supernatant was collected after homogenates were centrifuged at 12,000*g* for 10 minutes. Protein concentration in each sample was determined using a standard bicinchoninic protein assay (BCA) (Thermo Fisher Scientific), and all samples were diluted to a final concentration of 2 μg/μL. For immunoblotting, samples (20–30 μg) were separated on a 4% to 20% Criterion TGX precast protein gel (Bio-Rad Laboratories). Afterwards, proteins were transferred onto nitrocellulose membrane, stained with Ponceau S solution (Sigma-Aldrich), and placed in blocking solution for 1 hour. Membranes were incubated overnight at 4°C with primary antibodies listed in Supplemental Table 7. Blots were washed and incubated in the appropriate secondary antibody at room temperature. Luminol-based ECL reagent (Bio-Rad Laboratories) was applied for visualization. Finally, proteins were imaged using the ChemiDoc MP Imaging System (Bio-Rad Laboratories) and quantified using Image Lab software, version 6.1.0 (Bio-Rad Laboratories). All bands were normalized to their respective Ponceau prior to analysis.

### Cellular fractionation.

Nuclear and cytosolic proteins were isolated from approximately 20 mg of muscle as previously described (63). Briefly, samples were manually homogenized using a micro pestle in 200 μl of STM buffer supplemented with halt protease and phosphatase inhibitor cocktail (Thermo Fisher Scientific). Samples were then centrifuged at 800*g* for 15 minutes at 4°C. Supernatant was transferred into another tube for isolation of cytosolic proteins, while the pellet was resuspended in NET buffer and used for nuclear isolation.

### FISH combined with MBNL1 IF.

FISH-IF experiments were performed as previously described (6, 13). OCT-embedded samples were sectioned into 10 μm fixed in 3% PFA for 30 minutes at 4°C, washed with 1× PBS, and permeabilized in prechilled 2% acetone for 5 minutes at room temperature. Slides were then incubated in a prehybridization solution, followed by a hybridization solution at 35˚C for 2 hours. The hybridization solution contained a modified DNA probe complementary to 10 CUG repeats (IDT). Samples were then washed in a posthybridization solution at 45˚C for 30 minutes, followed by another wash in 1× SSC buffer. Slides were then blocked in 10% goat serum and incubated in primary antibody (1:1000; a gift from C.A. Thornton, University of Rochester Medical Center, Rochester, New York, USA) overnight at 4˚C. Following incubation, slides were washed and incubated with an Alexa Fluor secondary antibody (1:500; Thermo Fisher Scientific) and DAPI (1:20,000; Thermo Fisher Scientific). Finally, slides were dried, mounted using Prolong Gold Antifade (Thermo Fisher Scientific), and cover slipped. Slides were imaged using confocal microscopy (Nikon Instruments). Three 60× magnification Z-plane images and images of individual myonuclei were taken and used for analysis. Images were taken every 0.5 μm throughout the entire muscle cross section. Artificial CUG foci were detected in CON muscle due to nonspecific binding of the CUG probe. Therefore, the degree of MBNL1 sequestration was determined by counting the number of myonuclei (~142 per sample) with overlapping CUG and MBNL1 foci (+MBNL1/CUG myonuclei), which was rarely detected in CON muscle (<1%).

### RNA isolation and RNA-Seq.

RNA was extracted from approximately 5 to 10 mg of muscles using the QIAGEN miRNeasy Mini Kit (QIAGEN, 217004) as described by the manufacturers. Next-generation sequencing (NGS) libraries were created using the TruSeq Stranded Total RNA Library (Illumina) Kit. Sequencing was performed on an Illumina NovaSeq 6000 instrument. Alignment of FASTQ files was performed against the GRCh37 reference by HISAT2 (version 2.1.0) (64). Accurate reconstruction of all transcript isoforms was performed by StringTie (version 2.1.4) (65), with gene read count abundance determined by HTSeq (66). Differential expression analysis was performed by DESeq2 for library size normalization and statistical significance calculations while taking into account the group and paired sample study design (67). Pathway analysis was performed using iDEP (version 0.90) using the Go Biological Process gene ontology reference (68). Differential alternative splicing events and their significance were determined by rMATS (version 4.1.1) (69). Pilot extractions for a pair of DM1-PRE and DM1-POST samples (P6) were initially performed; the remaining DM1-PRE samples, DM1-POST samples, and CON samples were extracted in individual batches using identical extraction kits and techniques.

### Muscle snoRNA score.

A muscle snoRNA score calculation was implemented by calculating the total fraction of the counts of snoRNAs in each sample. Thirty-nine snoRNAs were identified from the top 500 differentially expressed genes between DM1-PRE and CON samples (Supplemental Table 3). All 39 snoRNAs were utilized for the final calculation (Supplemental Table 4). The raw counts associated with each of the snoRNAs were summed and the sum divided by the total count across all genes in each sample to provide a normalized total score.

### Preparation of permeabilized muscle fibers.

Immediately after biopsy collection, approximately 5 to 10 mg pieces of muscle were immersed in prechilled BIOPS solution. Under a light microscope, samples were stripped of connective tissue, blood, and fat using fine-tip forceps. Muscle fibers were then separated and divided into 2 roughly even bundles. Each bundle was washed on a rotating mixer for 30 minutes at 4°C in BIOPS solution that was treated with 40 μg/mL saponin. Finally, muscle bundles were washed in MiR05 prior to analysis.

### Mitochondrial respiration.

Mitochondrial respiration experiments were performed in 2 mL of MIR05 at 37°C using Oroboros Oxygraph-2k (Oroboros Instruments). MiR05 contained 5 μM blebbistatin and 20 mM creatine during all experiments. Each assay began with oxygenating each chamber to an O_2_ concentration of approximately 350 μM. After steady state was reached, respiration was stimulated with an ADP titration (25–8000 μM) in the presence of pyruvate (5 mM) and malate (2 mM). Glutamate (5 mM) was then added to measure complex I maximal respiration. Subsequently, succinate (20 mM) and rotenone (0.5 μM) were added to assess complex I+II and complex II maximal respiration, respectively. To ensure mitochondrial membrane integrity, cytochrome *c* (10 μM) was added to confirm less than 10 % change in respiration.

### Statistics.

The purpose of this trial was primarily to investigate exercise-induced clinical and physiological benefits in DM1 patients. Therefore, a 2-tailed, paired Student’s *t* test corrected for multiple comparisons was implemented to determine exercise changes in DM1 patients. An ordinary 1-way ANOVA with Bonferroni’s correction was employed to determine significance between CON participants and DM1 patients at the before and after time points. All statistical analysis was completed on GraphPad Prism software, version 9 (GraphPad Software). Statistical significance was defined as *P* < 0.05.

### Study approval.

This trial was approved by the Hamilton Integrated Research Ethics Board (no. 7091), complied with the guidelines set out in the Canadian Tri-Council policy statement on ethical conduct for research involving humans, and adhered to the 2013 World Medical Association adoption of the Declaration of Helsinki. All participants were informed of the nature and possible risks of the experimental procedures before their written, informed consent was obtained. All testing and experimental procedures were done after obtaining ethics approval and written, informed consent from each participant. This study was registered on clinicaltrials.gov (NCT04187482).

### Data availability.

RNA-Seq results were deposited in the NCBI’s Gene Expression Omnibus database (GEO GSE184951). The complete study protocol can be found in the Supplemental Methods. Individual patient clinical can be found in Supplemental Table 2, bulk RNA-Seq data can be found in Supplemental Table 3, and rMATS raw outputs can be found in Supplemental Table 5.

## Author contributions

AIM designed the trial, collected samples and data, performed experiments, interpreted results, and drafted the manuscript. PLN, NR, MFN, and PS performed RNA-Seq experiments. KM assisted with data collection and experiments. AM, SYN, and VL performed FISH-IF experiments. JQL performed histopathology examination. JPN assisted with interpreting results and drafting the manuscript. MAT designed the trial, collected samples and data, interpreted results, and assisted with drafting the manuscript. All authors approved the final version of this manuscript and agree to be accountable for all aspects of the work.

## Supplementary Material

Supplemental data

ICMJE disclosure forms

Supplemental table 2

Supplemental table 3

Supplemental table 4

Supplemental table 5

Supplemental table 6

## Figures and Tables

**Table 2:**
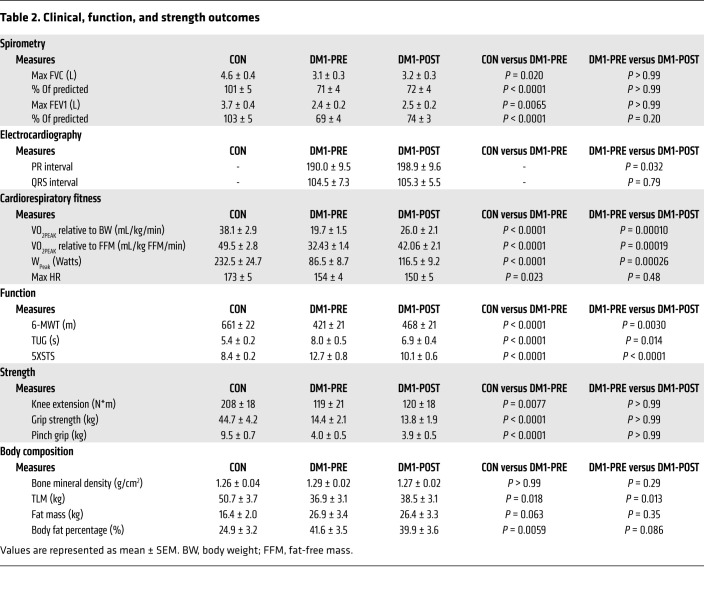
Clinical, function, and strength outcomes

**Table 1:**
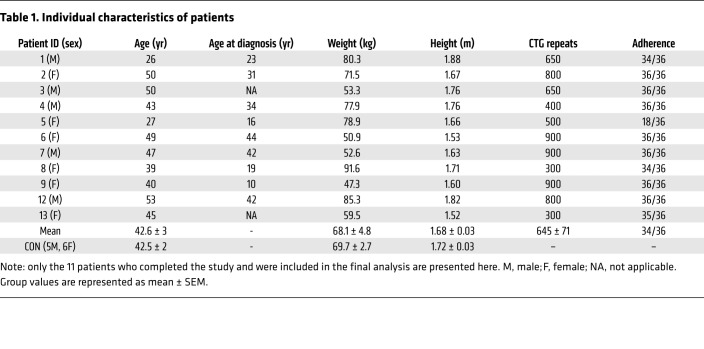
Individual characteristics of patients

## Version 1 03/22/2022

In-Press Preview

## Version 2 05/16/2022

Electronic publication
