# Supplementary material for: Aerobic exercise elicits clinical adaptations in myotonic dystrophy type 1 patients independently of pathophysiological changes
Source: J Clin Invest. 2022 May 16;132(10):e156125. doi: 10.1172/JCI156125 (PMC9106360; doi:10.1172/JCI156125)
Supplement: Supplemental data [file jci-132-156125-s235.pdf]

Title: Aerobic exercise elicits clinical adaptations in myotonic dystrophy type 1 patients independent of pathophysiological changes

Authors: Andrew I Mikhail[1], Peter L Nagy[2], Katherine Manta[3], Nicholas Rouse[2], Alexander Manta[1], Sean Y Ng[1], Michael F Nagy[2], Paul Smith[2], Jian-Qiang Lu[4], Joshua P Nederveen[3], Vladimir Ljubicic[1], Mark A Tarnopolsky*[3,5].

Affiliations:

[1]Department of Kinesiology, Faculty of Sciences, McMaster University, Hamilton, Ontario, Canada

[2]Praxis Genomics, LLC, Atlanta, Georgia, USA

[3]Department of Pediatrics, McMaster University Children's Hospital, Hamilton, Ontario, Canada

[4]Department of Pathology and Molecular Medicine/Neuropathology, McMaster University, Hamilton, Ontario Canada

[5]Exerkine Corporation, McMaster University Medical Center, Hamilton, Ontario, Canada

*To whom correspondence should be addressed:

Dr. Mark A Tarnopolsky

1200 Main Street West Hamilton, Ontario, Canada, L8N3Z5

Phone: (905)-577-2100 ext 75226

E-mail: tarnopol@mcmaster.ca

Table S1: Blood work

Measures	DM1-PRE	DM1-POST	
GLUF (mmol/L)	5.1 ± 0.07	5.4 ± 0.09	$P < 0.05$
CK (units/L)	240.7 ± 44.5	290.0 ± 49.6	NS
Creatinine (μmol/L)	71.6 ± 3.4	74.1 ± 4.9	NS
Bilirubin (μmol/L)	8.4 ± 0.7	8.5 ± 1.0	NS
ALT (units/L)	39.3 ± 9.0	52.4 ± 12.2	NS
GTT (units/L)	94.9 ± 36.6	75.0 ± 22.1	NS

GLUF, fasting blood glucose. CK, creatine kinase. ALT, alanine transaminase. GTT, gamma-glutamyl transpeptidase. Values are mean ± SEM.

Table S7: List of primary antibodies

Western Blotting					
**Protein**	**Manufacture**	**Product #**	**Host**	**Primary Dilution**	**Secondary Dilution**
AMPK	Cell Signaling Technology	2532	Rabbit	1:1000	1:20,000
pAMPK	Cell Signaling Technology	2531	Rabbit	1:1000	1:20,000
BNIP3	Cell Signaling Technology	3769	Rabbit	1:1000	1:20,000
CUGBP1	Santa Cruz Biotechnology	sc-20003	Mouse	1:1000	1:20,000
DRP1	Cell Signaling Technology	8570	Rabbit	1:1000	1:20,000
p-DRP1^{S616}	Cell Signaling Technology	3455	Rabbit	1:1000	1:20,000
p-DRP1^{S637}	Cell Signaling Technology	4867	Rabbit	1:1000	1:20,000
Fis1	Proteintech	10956-1-AP	Rabbit	1:1000	1:20,000
GAPDH	ThermoFisher Scientific	PA1-987	Rabbit	1:1000	1:20,000
GSK-3β	Cell Signaling Technology	9315	Rabbit	1:10,000	1:20,000
pGSK-3β	Cell Signaling Technology	9323	Rabbit	1:10,000	1:20,000
Histone H3	Abcam	ab18521	Rabbit	1:1000	1:20,000
MBNL1	Abnova	H00004154-M02	Mouse	1:1000	1:20,000
MBNL2	Santa Cruz Biotechnology	sc-136167	Mouse	1:1000	1:20,000
MFN-1	Cell Signaling Technology	14739	Rabbit	1:1000	1:20,000
MFN-2	Abcam	ab56889	Mouse	1:1000	1:20,000
OPA1	Abcam	ab42364	Rabbit	1:1000	1:20,000
OXPHOS	Abcam	ab110413	Mouse	1:1000	1:20,000
Parkin	Cell Signaling Technology	2132	Rabbit	1:1000	1:20,000
PGC-1α	EMD Millipore	ST1202	Mouse	1:1000	1:20,000
PINK1	Novus Biologicals	BC100-494	Rabbit	1:1000	1:20,000
**Immunofluorescence**					
**Protein**	**Manufacture**	**Product #**	**Host**	**Primary Dilution**	**Secondary Dilution**
MHC I	DHSB	BA-F8	Mouse (IgG2b)	1:100	1:500
MHC IIA	DHSB	SC-71	Mouse (IgG1)	1:600	1:500
MHC IIX	DHSB	6H1	Mouse (IgM)	1:25	1:500
Wheat Germ Agglutinin	Thermo Fisher Scientific	W11263	Conjugated	1:300	NA

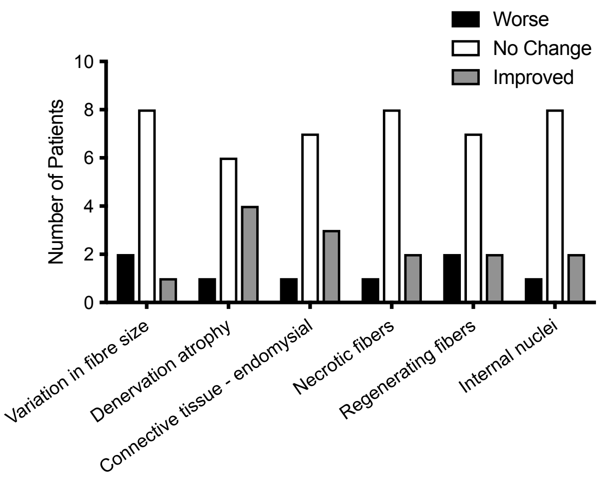

Supplementary figure 1. A blinded histopathological examinations of skeletal muscle samples from the *vastus lateralis* of DM1 patients for metrics of muscle damage before and after exercise training. Worse pathology scores after exercise training are indicated in black bars, no change in pathology scores after exercise are indicated in white bars and improved pathology scores after exercise are indicated in grey bars. n = 11.

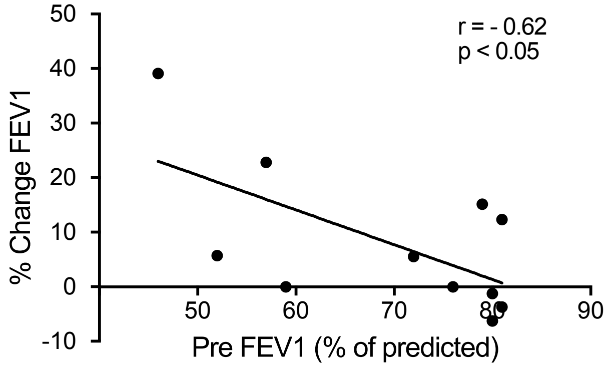

Supplementary figure 2. A Pearson correlation analysis between baseline forced expiratory volume (FEV1), made relative to predicated values, and percent change in FEV1 values before and after exercise. n = 11.

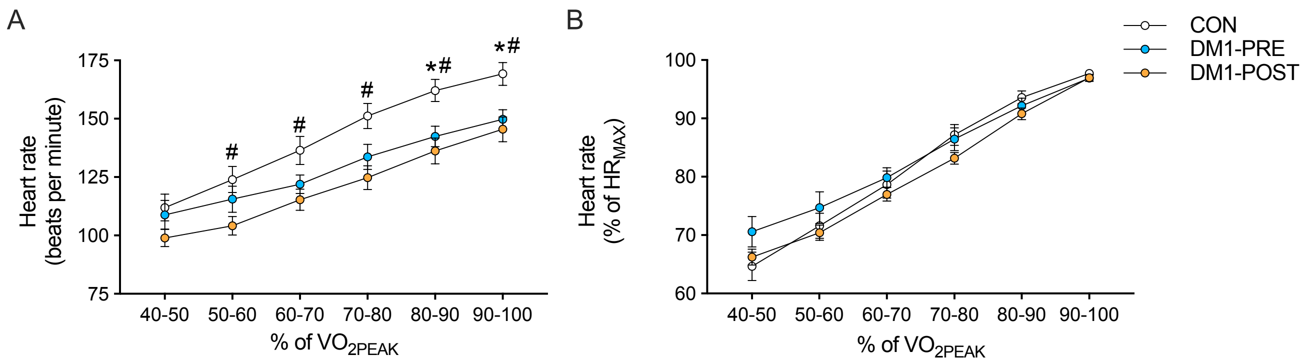

Supplementary figure 3. (A) Absolute and **(B)** relative heart rate in response to a progressive increase in exercise intensity. Data are expressed as mean ± SEM. n = 11. * $p < 0.05$ CON vs DM1-PRE and # $p < 0.05$ CON vs DM1-POST; One-way ANOVA followed by a Bonferroni correction.

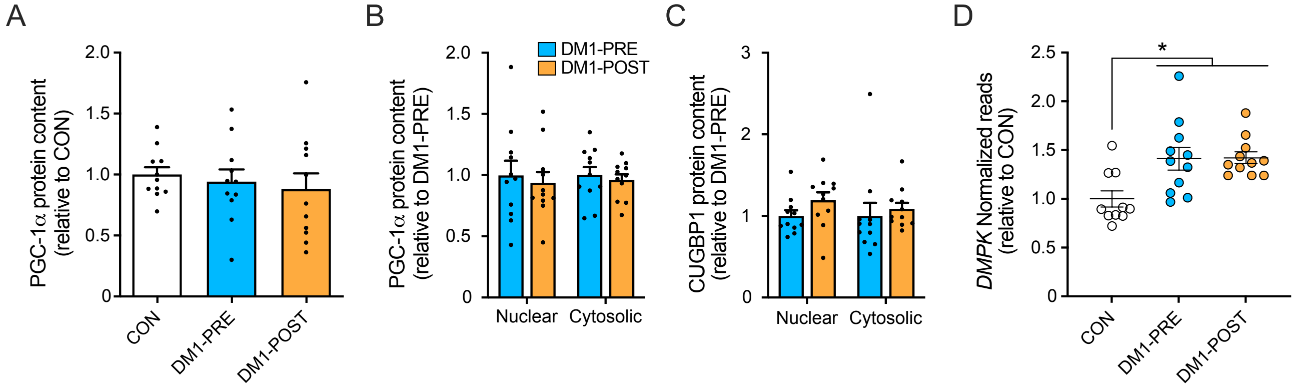

Supplementary figure 4. Graphical summary of **(A)** whole lysate peroxisome proliferator-activated receptor gamma coactivator 1-alpha (PGC-1α) as well as nuclear and cytosolic **(B)** PGC-1α and **(C)** CUGBP1 in the *vastus lateralis* muscle. **(D)** Normalize reads of total *DMPK* mRNA expression in healthy controls and DM1 participants before and after exercise. Data are expressed as mean ± SEM with individual data points. n = 11. * $p < 0.05$ vs CON; One-way ANOVA followed by a Bonferroni correction. # $p < 0.05$ vs DM1-PRE; two-tail paired t-test corrected for multiple comparisons.

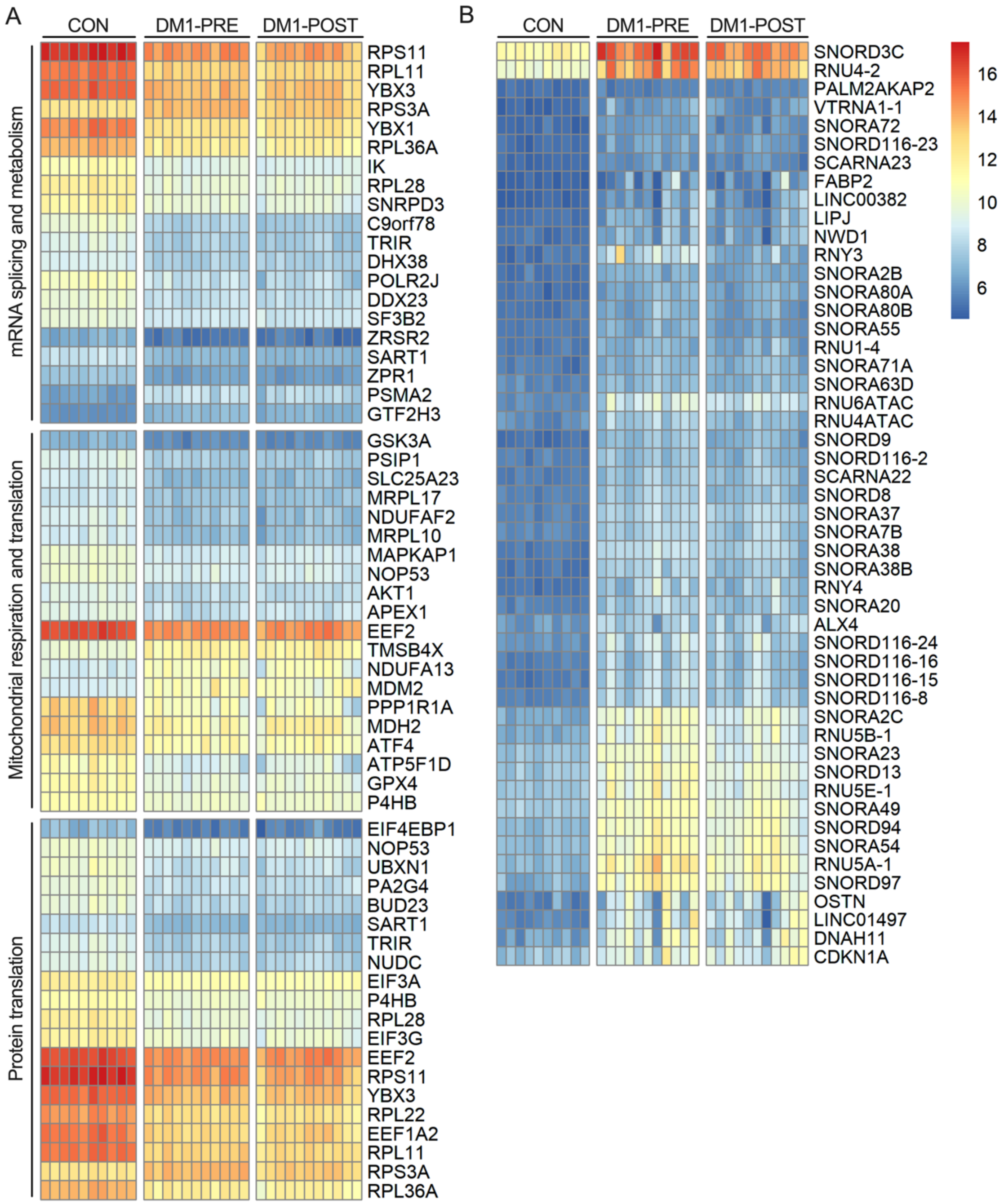

Supplementary figure 5. (A) Top differentially expressed genes within downregulated pathways relating to mRNA splicing and metabolism, mitochondrial respiration and translation, and protein translation. **(B)** Heat map of the top 50 upregulated genes in DM1-PRE relative to CON participants. n = 10 – 11.

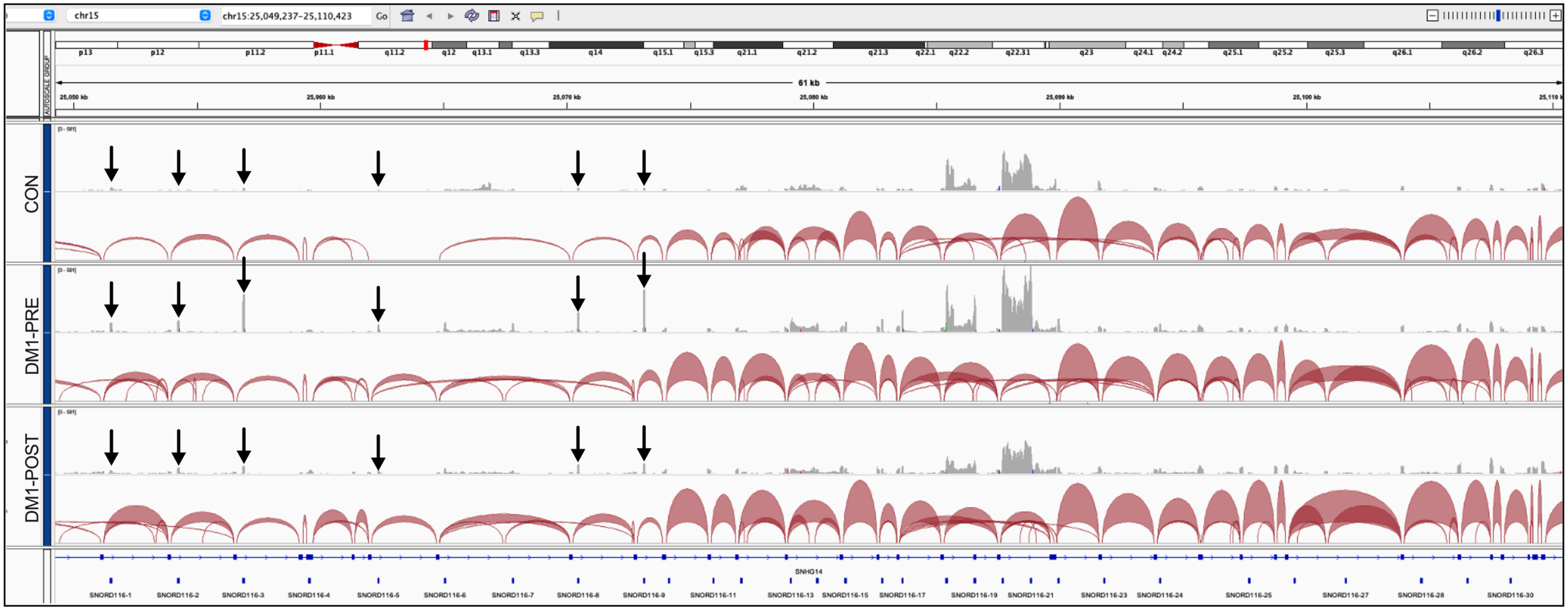

Supplementary figure 6. Read coverage (top) and splice junction (bottom) tracks for *small nucleolar RNA host gene 14* (*SNHG14*) in CON, DM1-PRE and DM1-POST muscle samples. Black arrows highlight difference in read coverage for several snoRNAs that reside within *SNHG14*.

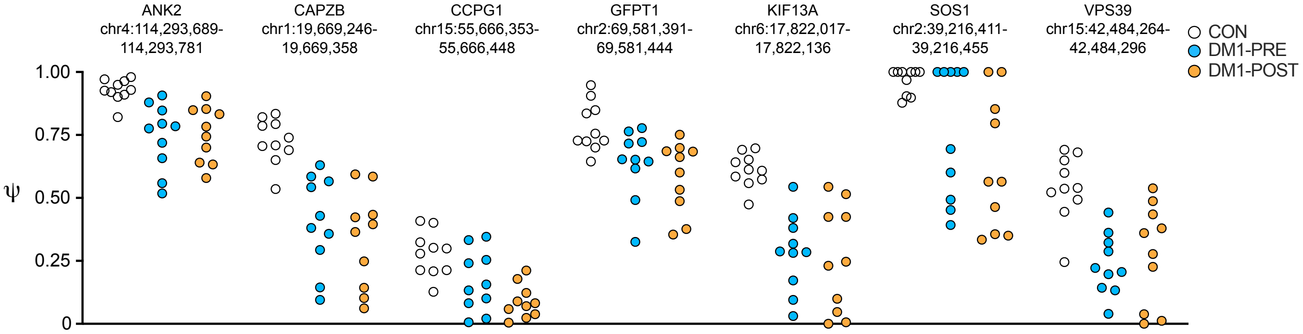

Supplementary figure 7. Scatterplot of individual percent spliced in (PSI or ψ) values for canonical missplicing events in DM1 biology. Samples are from the *vastus lateralis* muscle of healthy controls (CON), and DM1 patients before (DM1-PRE) and after (DM1-POST) exercise. n = 10.

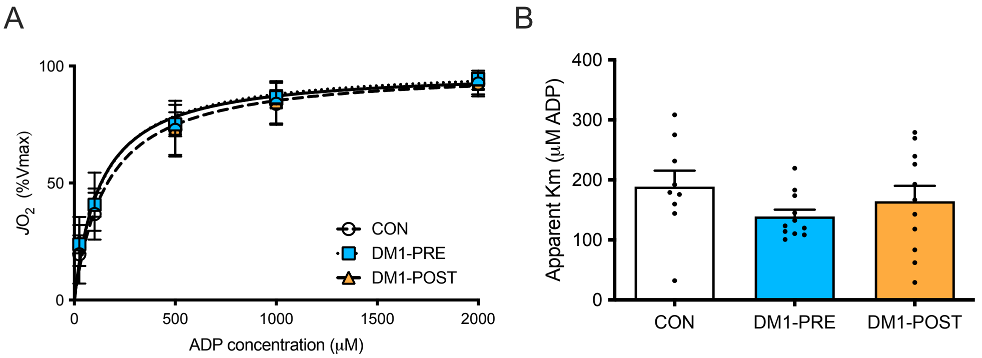

Supplementary figure 8. (A) Michaelis-Menten kinetic analysis to derive a predicted value of ADP sensitivity for healthy controls (CON), DM1 patients before exercise (DM1-PRE) and after exercise (DM1-POST). **(B)** Graphical summary of the apparent Km for CON, DM1-PRE and DM1-POST. Data are expressed as mean ± SEM. n = 9 – 11.

SUPPLEMENTAL METHODS

Study Title: Functional and cellular benefits of aerobic exercise in myotonic dystrophy type 1 patients

Principal Investigator: Mark Tarnopolsky M.D., PhD[1]

Institution: [1]Department of Medicine and Pediatrics, McMaster University, Neuromuscular and Neurometabolic Clinic and Research

Funding Source: Canadian Institutes of Health Research (CIHR)

Table of Contents

1. STUDY BACKGROUND 4

2. OBJECTIVES 5

3. TARGET POPULATIONS 5

4. INCLUSION CRITERIA 5

5. EXCLUSION CRITERIA 6

6. RECRUITMENT PROCEDURE 6

7. SAMPLE SIZE ESTIMATE 6

8. STUDY DESIGN 7

8.1. GENERAL EXPERIMENTAL DESIGN 7

9. CONFLICT OF INTEREST RESOLUTION CLAUSE 8

10. PROCEDURES 9

10.1. *DUAL X-RAY ABSORPTIOMETRY (DXA) AND BLOOD PRESSURE* 9
10.2. *WAIST AND HIP CIRCUMFERENCE MEASURES* 9
10.3. MAXIMAL VOLUNTARY VO_{2PEAK} FITNESS TEST 9
10.4. *ELECTROCARDIOGRAM (ECG)* 9
10.5. *SPIROMETRY* 10
10.6. *MAXIMAL VOLUNTARY HAND GRIP ASSESSMENT* 10
10.7. *MAXIMAL VOLUNTARY KNEE EXTENSION STRENGTH TEST* 10
10.8. *QUESTIONNAIRES* 10
10.9. *FUNCTIONAL TESTING* 10
10.10. *BLOOD SAMPLING* 10
10.11. *URINE SAMPLING* 11
10.12. *MUSCLE SAMPLING* 11

11. STATISTICAL ANALYSIS 11

12. COMPENSATION 11

13. ADVERSE EVENTS 11

1. Study background

Myotonic dystrophy type 1 (DM1) is the second most common muscular dystrophy after Duchenne muscular dystrophy (DMD). DM1 has a prevalence rate ~1/500 in some parts of Quebec and affects ~1/8000 individuals worldwide making it the most prevalent form of adult muscular dystrophy [1,2]. It often presents as a neuromuscular condition with hypotonia in young children and distal muscle atrophy and weakness later in life. In addition to the effect on the musculoskeletal system, DM1 is a multi-systemic disorder with co-morbidities including insulin resistance (dysglycemia)[3], cardiac conduction and rhythm defects[4], cataracts[5], gonadal atrophy[6] and gastrointestinal[7] and neuropsychiatric[8] symptoms. Consequently, individuals with DM1 can appear hypersomnolent[9] and have significant functional limitations which impair their ability to perform activities of daily life and exercise. The progressive nature of the disease forces DM1 patients to be wheelchair-bound or require the use of a walking aid as a result of accelerated muscle weakness and wasting. The resultant inactivity and sedentary lifestyle, secondary to the functional limitations imposed by muscle wasting may further exacerbate metabolic risk factors such as dysglycemia[10]. With no current cure, much of the research has focused on strategies that would mitigate muscle wasting while reducing secondary co-morbidities. Endurance training has the potential to show considerable benefits for patients with DM1 by providing an anabolic stimulus to promote muscle strength and improving insulin resistance and fatigue resistance.

DM1 arises from an autosomal dominant trinucleotide repeat mutation and results in a multisystemic neuromuscular disease. It is caused by a cytosine-thymine-guanosine (CTG) repeat expansion in the 3’ untranslated region of the dystrophia myotonica protein kinase (*DMPK*) gene[11,12]. Healthy individuals possess 5-37 repeats; however, the DM1 phenotype begins to manifest in individuals with over 50 repeats[13]. The resultant RNA transcribed from *DMPK* gene with CTG repeats are rich in CUG repeats which are highly stable and resistant to degradation due to the presence of secondary hairpin structures[14]. These toxic, mutant RNA species are retained in the nucleus forming nuclear foci[15]. Nuclear retention of these mutant RNA species disrupts the function of two RNA-binding proteins; muscleblind-like 1 (MBNL1) and CUG-binding protein 1 (CUGBP1)[16–18]. The primary consequence of RNA toxicity is the dysregulation of alternative splicing (spliceopathy) producing an impaired ability to process RNA species that require post-transcriptional modification (alternative splicing)[19]. The DM1 phenotype cannot be explained by DMPK deficiency alone[20], indicating that toxic RNA-mediated spliceopathy has collateral effects on additional transcripts (chloride channels[21], insulin receptor[22], dystrophin[23]). As such, at least one dozen genes have been identified to be affected due to spliceopathy in DM1.

A recently published review has outlined the possible role and benefits of 5' adenosine monophosphate-activated protein kinase (AMPK) in various neuromuscular diseases including DMD, spinal muscular atrophy and DM1[24]. In context of DM1, pharmacological activation of AMPK in DM1 patients using metformin administration was found to be an effective treatment for attenuating insulin resistance[25]. Furthermore, AICAR, another potent activator of AMPK, was able to correct splicing of various transcripts in DM1 cells[26] and significantly reduced myotonia in DM1 mice[27]. Endurance exercise training is a natural physiological activator of AMPK that elicits favourable muscle and multisystemic benefits[28]. Pre-clinical trials looking at

the effect of exercise training on DM1 found that exercise was able to correct splicing of various mis-spliced genes[29,30], improve strength and functional ability, liberate MBNL1, and reduce myotonia in a mice model of DM1[30]. More importantly, endurance exercise training in DM1 patients improved cardiorespiratory fitness and peak power output[31,32], as well as increased muscle fibre cross-sectional area (a measure of hypertrophy)[32]. To date, little is known about the potential benefits of exercise on function and strength in DM1 patients[33]. Additionally, current literature is lacking evidence for potential cellular mechanism through which exercise could improve the DM1 phenotype.

2. Objectives

Our primary aim is to determine the influence of moderate intensity aerobic exercise on clinically relevant outcomes such as 6-minute walk test, body composition (i.e., fat mass loss, lean mass preservation), and strength when administered over a 12-week period. Secondarily to this, we will determine how mitochondrial function/content, alternative splicing of genes critical to muscle health and CUG repeats are affected by this intervention.

VO_{2peak} will be used as our primary outcome to measure changes in cardiorespiratory fitness before and after exercise. Our secondary outcomes will include 6-minute walk test, 5x sit to stand, timed up and go, maximal voluntary knee extension and maximal voluntary grip strength. Finally, to determine any possible exercise-induced cellular benefits as previously shown in murine models[29,30], we will measure mitochondrial function, splicing of various genes (chloride channels and insulin receptors) and MBNL1 sequestration as exploratory measures.

3. Target populations

The present study will target patients (men and women) that have been clinically diagnosed with myotonic dystrophy type 1 between the ages of 18 – 60 with 100-1000 CTG repeats. We expect this population to be physically inactive (e.g., < 1 hour of formal exercise/week).

4. Inclusion criteria

In order to participate in the study, volunteers must satisfy <u>all</u> of the following criteria:

1. Myotonic Dystrophy type 1 subject inclusion criteria:
   - Male or female clinically diagnosed with DM1 (age 18 – 60 y).
   - CTG repeats 100-1000.
   - Normal weight (BMI 18.5 – 24.9 kg/m^2) or overweight (BMI 25 – 29.9 kg/m^2).
   - Physically inactive (< 1 hour of formal exercise/week).
   - 6-minute walk test score between 250 – 500 meters
   - ECG with PR interval < 225 ms and QRS duration < 125 ms.

2. Age and sex-matched controls:
   - Healthy men and women aged 18 – 60 y.
   - Normal weight (BMI 18.5 – 24.9 kg/m^2) or overweight (BMI 25 – 29.9 kg/m^2)
   - Physically inactive (< 1 hour of formal exercise/week)

5. **Exclusion criteria**

Participants will be excluded and/or released from the study if they meet any one of the following: Smoking, obese (BMI > 30.0 kg/m^2), physically active (> 1-2 hour of formal exercise/week), 6-minute walk test score <250 meters, chronic (> 2 weeks) use of narcotic analgesic or anti-inflammatory drugs, type 1 or 2 diabetes (more than one anti-diabetic drug), cardiovascular disease (recent myocardial infarction (< 6 months)), uncontrolled hypertension requiring more than 2 medications, congestive heart failure requiring more than one medication for control, cardiac conduction block (as above); renal disease (creatinine > 140), known liver disease, cognitive impairments limiting ability to provide informed consent, previous stroke with residual hemiparesis, active musculoskeletal injuries and/or severe osteoarthritis, significant weight loss in the 3-month period prior to the study, vegan diet, dairy protein allergy, severe peripheral neuropathy, severe osteoporosis, use of medications known to affect protein metabolism (i.e. corticosteroids), chronic obstructive or restrictive pulmonary disease (FVC < 70% of age predicted mean value), or asthma requiring more than two medications. Participants on volitional dietary supplements will be considered on a case-by-case basis, but they will be asked to refrain from intake for at least 2 weeks prior to partaking in this study. Although we do not anticipate any adverse events, participants will be asked to disclose if they are using supplements that may have negative/interactive effects with our study protocol.

6. **Recruitment Procedure**

A total of 18 men and women diagnosed with DM1 will be notified of the study through Dr. Mark Tarnopolsky, who will introduce the study to eligible DM1 patients and leave the explanation of the study, recruitment and consent form signing to the study coordinator. 18 healthy men and women will be recruited via passive means including the dissemination of HiREB-approved newspaper advertisements and posters at gathering locations. During the initial contact (phone or personal interview) with a potential participant, a study coordinator will provide a brief and simple overview of the study. Following an overview, the criteria for inclusion and exclusion will be outlined and discussed. Those who qualify will be invited to the Neurometabolic Clinic in the Department of Pediatrics located in McMaster Children's hospital. There, one of the coordinators will describe the study. During this interaction, potential participants will be required to read the Information and Consent Form, and all procedures will be fully described. A written copy of the consent form will be provided for the participants to read by themselves. Written informed consent will then be obtained.

7. **Sample size estimate**

For many of our outcome variables we do not have *a priori data* in DM1 patients and we will consider a differential improvement of ≥ 30 % in most of these to be potentially clinically relevant and worthy of further investigation. However, previous evidence suggests ~14% (4.8 ml/kg/min) increased cardiorespiratory fitness in response to a similar exercise protocol as the currently proposed study[32]. Using this published study, we calculate the sample size to be:

Sample size = $2SD^2$(Z table at type 1 error of 5% [1.96] +Z table at 80% power [0.84])$^2 \div d^2$
SD (Standard deviation) = 4.6 ml/kg/min

d (Mean difference) = 37.3 ml/kg/min (post-exercise) – 32.5 ml/kg/min (pre-exercise) = 4.8 ml/kg/min
$2(4.6)^2(1.96 + 0.84)^2 \div (4.8)^2 = \sim 14$/group.

Additionally, based on Dr. Tarnopolsky's clinical expertise and previous studies of a similar nature, we anticipate a drop-out rate of ~30%. Therefore, we intend to recruit 18 subjects per group.

8. Study design

The present study is a repeated measures design, with sampling of the cohorts on two separate occasions. Upon obtaining informed consent, participants will be assigned into one of two groups:

Group 1: DM1 Exercise Group
DM1 diagnosed, physically inactive males, n = 9
DM1 diagnosed, physically inactive females, *n* = 9

Group 2: Healthy controls (no exercise)
Healthy, physically inactive males n = 9
Healthy, physically inactive females n = 9

8.1. General experimental design

Introduction – Day 1 (Visit 1); On the initial visit, the potential participant will come to McMaster Children's Hospital to meet with a study coordinator involved in the project. The coordinator will explain the study in detail, answer any questions, and review the consent form. Upon obtaining informed consent, eligible participants will complete a medical screening questionnaire to determine their readiness to perform exercise. Participant characteristics will then be measured. At this time, anthropometric measurements (i.e., height, weight, waist circumference) and a dual energy x-ray absorptiometry (DXA) measurement will be performed. This must be done in order to verify that the participant meets the inclusion criteria of being normal weight (BMI 18.5 – 24.9 kg/m^2) or overweight (BMI 25 – 29.9 kg/m^2). Following this screening, participants will undergo an ECG to measure any conductance blockage and perform a maximal cycling test to determine their aerobic capacity ($V0_{2peak}$). Maximal aerobic testing will then be followed by another 12 lead ECG to detect any possible structural cardiac issues in our participants. Thereafter, participants will begin the following the study timeline, all of which will occur at the McMaster Children's Hospital, 2H Neurometabolic Clinic.

Baseline testing – Day 3 (Visit 2); Participants will arrive the following day to undergo a spirometry test, functional testing which will include 6-minute walk test, 5x sit to stand, timed up and go, grip strength and maximal voluntary knee extension using the Biodex dynamometer. Participants will then complete the SF-36.

Follow up testing – Day 5 (Visit 3); Participants will arrive following an overnight fast (including no caffeine for 12 hours) and having abstained from any exercise for the prior 24 hours. Participants will undergo a muscle biopsy from the *vastus lateralis*. Blood will be drawn

from an antecubital vein. Finally, participants will be asked to give a urine sample in a fasted state.

DM1 participants only:

Exercise training – Visit 4 – 39; Participants will begin the exercise protocol consisting of 3 exercise sessions per week for a 12-week period. All exercise sessions will be done on a cycle ergometer (Lode, Groningen, Netherlands). Each exercise session will consist of 3 minutes of warm up, 30 minutes at 65% $V0_{2peak}$ and will end with 2 minutes of cool down. Exercise progression over the 12 weeks will go as follows:

- Weeks 1 and 2: 3 min warm up, 65% $VO2_{max}$ for 30 min, 2 min cool down
- Weeks 3 and 4: increase to 35 min (+ warm up/cool down)
- Weeks 5 and 6: drop to 30 min and increase to 70% VO2max
- Weeks 7 and 8: increase to 35 min (+ warm up/cool down)
- Weeks 9 and 10: drop to 30 min and increase to 75%VO2 max
- Weeks 11 and 12: increase to 35 min (+ warm up/cool down)

Endpoint testing – Visit 40; At this time, anthropometric re-measurements (i.e., height, weight, waist circumference) and a dual energy x-ray absorptiometry (DXA) measurement will be performed. Thereafter, participants will undergo an ECG to measure any conductance blockage and perform a maximal cycling test to determine their aerobic capacity ($V0_{2peak}$). Maximal aerobic testing will then be followed by another 12 lead ECG to detect any possible structural cardiac issues in our participants.

Endpoint testing – Visit 41; Participants will arrive the following day to undergo a spirometry test, functional testing which will include 6-minute walk test, timed up and go, grip strength and maximal voluntary knee extension using the Biodex dynamometer. Participants will then complete the SF-36.

Endpoint testing – Visit 42; Participants will arrive following an overnight fast (including no caffeine for 12 hours) and having abstained from any exercise for the prior 24 hours. Participants will undergo a muscle biopsy from the *vastus lateralis*. Blood will be drawn from an antecubital vein. Finally, participants will be asked to give a urine sample in a fasted state.

9. Conflict of Interest Resolution Clause

DM1 participants will be notified of the study through the Neuromuscular and Neurometabolic clinic by Dr. Mark Tarnopolsky. However, Dr. Tarnopolsky will not be involved in the recruitment process and all participants will be recruited by research staff in our laboratory. Following confirmation eligibility and clinical confirmation of DM1, only research staff (Mr. Andrew Mikhail and Mrs. Kristin Barnard) will be taking consent from all participants, giving them further details of the study protocol, training participants and completing data collection.

10. Procedures

10.1. Dual X-ray absorptiometry (DXA) and blood pressure

A DXA scan is a non-invasive procedure requiring the participant to only lay still on a padded table. It is a fast procedure that provides a variety of body composition variables (bone mass/area/density, fat mass, and fat free mass). The DXA scan exposes individuals to low levels of radiation, approximately 0.18 millirem. According to the United States Nuclear Regulatory Commission, Americans receive approximately 620 millirem each year, half of which is due to environmental background radiation. Radiation exposure can be due to medical treatments or procedures, like X-Ray or MRI's, food, sun exposure, etc. Any exposure to radiation can increase the risk of cancer. Women of child bearing age who are pregnant, think they may be pregnant, or trying to become pregnant are advised to not undergo medical treatments that can expose them to radiation. The participants will be asked to notify the technician if they think they may be pregnant before having the DXA scan. In the present study, body composition will be assessed using DXA scan (GE Lunar Prodigy, Madison, WI) and a software program for adults (encore Version 9.15.010). Fat free mass (FFM), fat mass (FM) and bone mineral density (BMD) of the entire body will be recorded. Arterial blood pressure (i.e., systolic [SBP] and diastolic [DBP]) will be measured during rest with a stethoscope (Marshall Nurse Stethoscope, Riverside, Ill., USA) and sphygmomanometer (MDF Instruments Direct Inc., Agoura Hills, Calif., USA).

10.2. Waist and hip circumference measures

For waist circumference measurement, the participant will stand with arms at the sides, feet together, and abdomen relaxed. Using a Gulick tape measure, a horizontal measure will be taken at the narrowest part of the torso (above the umbilicus and below the xiphoid process). Obtaining a horizontal measure directly above the iliac crest enhances standardization according to The National Obesity Task Force. For hip circumference measurement, the participant stands erect and feet together, a horizontal measure is taken at the maximal circumference of buttocks.

10.3. Maximal voluntary VO_{2peak} fitness test

Participants will complete double-leg incremental peak oxygen uptake (VO_{2peak}) tests on a cycle ergometer (Lode, Groningen, Netherlands). A metabolic cart with an on-line gas collection system (Moxus modular oxygen uptake system, AEI Technologies, Pittsburgh, PA) will acquire oxygen consumption (VO_2) and carbon dioxide (CO_2) production data. For double-leg cycling, the test will begin with a 1-min warm up at 50 watts (W), after which the power will be increased by 1 W every two seconds until volitional exhaustion or the point at which pedal cadence falls below 60 rpm. Heart rate will be monitored continuously throughout the test via telemetry with a heart rate monitor (Polar A3, Lake Success, NY). VO_{2peak} will be defined as the highest oxygen consumption achieved over a 30-s period. Maximal workload (Wmax) will be the highest power output achieved during the test. After the completion of the test, the participant will be asked to perform a 2-3-minute active recovery pedaling against no resistance.

10.4. Electrocardiogram (ECG)

A standard 12-lead ECG are recorded immediately prior to and after performing VO_{2Peak} test at baseline and after exercise training period (DM1 only). Participants will be resting in a supine

position for at least 10 minutes using an electrocardiographic device. We will assess heart rate, rhythm, Interval from the beginning of the P wave until the beginning of the QRS complex (PR interval), and QRS duration.

10.5. Spirometry

Sitting comfortably upright, in ambient conditions, subjects will perform a series of 3 strong inhalations, followed by a fast a strong exhalation. Forced vital capacity and forced expiratory volume will be measured (in litres) using Sentry Suite version 2.11.

10.6. Maximal voluntary hand grip assessment

Hand grip strength will be measured using an isometric dynamometer (JAMAR®, Sammons, Bolingbrook, IL). The grip width will be adjusted to hand size and with arm flexed at 90°, the participant will perform three * 5 s efforts with a one min rest between trials.

10.7. Maximal voluntary knee extension strength test

Isometric knee extension will be measured by mechanical dynamometry (Biodex System 3, Biodex Medical Systems, Shirley, NY). Participants will be positioned into the machine with the knee flexed at 90° and perform three X 5s maximal voluntary contractions with 30s rest between each trial.

10.8. Questionnaires

The RAND 36-Item Health Survey (Version 1.0) taps eight health concepts: physical functioning, bodily pain, role limitations due to physical health problems, role limitations due to personal or emotional problems, emotional well-being, social functioning, energy/fatigue, and general health perceptions. It also includes a single item that provides an indication of perceived change in health.

10.9. Functional testing

A total of four functional tasks will be performed once using a stopwatch that records times to an accuracy 1/100 of a second. First, the 6-minute walk test will involve participants walking as fast as possible around a 20m track for 6 minutes and distance will be measured to the closest meter. Next, 5x sit-to-stand test will involve participants performing a series of consecutive rising and sitting positions from a sturdy, armless plastic chair secured against a wall and with arms crossed at the chest. Finally, the chair rise-and- walk test will involve participants starting from a seated position, standing up and walking as quickly as possible in a predetermined straight line to a pylon 9.14m, while going around the pylon, and returning to their original seated position.

10.10. Blood sampling

A total of 16 mL blood per visit will be taken from the antecubital vein and drawn into evacuated tubes with heparin used for plasma collection and non-treated tubes will be used to collect serum. Blood sampling will be used for the following panel of tests: complete blood count (CBC), glucose, Gamma-Glutamyl Transferase (GGT), bilirubin, Alanine Aminotransferase (ALT), creatine kinase (CK), creatinine, triglycerides, total cholesterol, LDL and HDL, insulin, CRP.

10.11. Urine Sampling

A total of 60 mL of urine will be collected in a fasted state, frozen in liquid nitrogen then stored in our laboratory (4N68) in -80 degree C freezer. Urine sampling will be used to measure CUG repeats before and after exercise intervention to measure potential multisystemic effects of exercise.

10.12. Muscle Sampling

Participants will arrive in the morning in the fasted state and rest quietly in the supine position for 10 minutes. A muscle biopsy will then be taken from the vastus lateralis using local anaesthetic as described[34]. Samples will be dissected free of connective tissue and immediately partitioned for subsequent analysis (~ 25 mg) for RNA, a small piece (~ 10 mg) will be placed into chilled glutaraldehyde for subsequent electron microscopic evaluation, ~ 20 mg will be placed in optimal cutting temperature (OCT) solution for cross-sectional analysis, and ~ 50 mg will be frozen for enzyme and protein analysis. Mitochondrial content and function will be examined by measuring protein and enzyme activity of different mitochondrial complexes (citrate synthase, etc..) using western blots and enzyme activity assays respectively. Furthermore, expression of different genes such as chloride channels and insulin receptor will be assessed using PCR to determine mRNA content. Lastly, muscleblind-like 1 sequestration will be assessed using western blots and fluorescence in situ hybridization.

11. Statistical analysis

The differences between conditions will be analyzed using repeated measures two-way analysis of variance (ANOVA) using between and within factors to test main effects and interactions. Main effects will be groups (between groups: DM1 vs. healthy controls) and exercise (pre- and post-exercise period). Tukey's HSD post hoc testing will be used for multiple comparisons. Statistical significance will be accepted at $\alpha<0.05$. SPSS (version 21; SPSS, Inc., Chicago, IL) will be used for all statistical analyses.

12. Compensation

Participants completing the full study will be given $350 to compensate for their time and any additional expenses they may incur as part of the study (i.e., parking if necessary, per visit). For those completing visits 1,2 and 3 only, they will receive a maximum of $150 for their involvement. In the event that you cannot complete the requirements of the study, you will receive a pro-rated amount at the rate of $50 per session, to a maximum of $350.

13. Adverse events

Participants will be asked to supply a sample of muscle and venous blood. An experience individual who has obtained the required training will perform the muscle biopsy and blood draw. In the very unlikely event that the participant feels ill (i.e., light-headedness), precautions can be taken with physicians and/or nurses present. If symptoms are present, we will ask the participant to stay seated and monitor their blood pressure to ensure they are not at risk for syncope. If light-headedness persists, we may give the participant a sugary drink (e.g. orange juice). Only once the participant is feeling better will we proceed with testing. If the condition worsens, Dr. Tarnopolsky will direct.

Participants will also be asked to perform a maximal voluntary fitness test. To minimize risks, only trained technician will be performing this procedure. In addition, participants' will have their heart rate monitored continuously throughout the test. The test will be discontinued if any abnormal heart rate or rhythm is detected, in addition to chest pain, light-headedness, and vertigo. Should these symptoms arise, Dr. Tarnopolsky will immediately assess the situation and if required call 5555 to access the hospital's Advanced Cardiovascular Life-support System (ACLS) team or other emergency services.

14. References

1. Chau, A. & Kalsotra, A. Developmental insights into the pathology of and therapeutic strategies for DM1: Back to the basics. *Dev. Dyn.* **244**, 377–390 (2015).
2. Harley, H. G. *et al.* Expansion of an unstable DNA region and phenotypic variation in myotonic dystrophy. **355**, 1991–1992 (1992).
3. Matsumura, T. *et al.* Journal of the Neurological Sciences A cross-sectional study for glucose intolerance of myotonic dystrophy. *J. Neurol. Sci.* **276**, 60–65 (2009).
4. Bassez, G. *et al.* Severe cardiac arrhythmias in young patients with myotonic dystrophy type 1. 13–16 (2004).
5. Reardon, W. *et al.* Cataract and myotonic dystrophy : the role of molecular diagnosis. 579–583 (1993).
6. Al-harbi, T. M., Bainbridge, L. J., Mcqueen, M. J. & Tarnopolsky, M. A. Hypogonadism is Common in Men With Myopathies. 3–7 (2008).
7. Bellini, M. *et al.* Gastrointestinal manifestations in myotonic muscular dystrophy. **12**, 1821–1828 (2006).
8. Ekström, A.-B., Hakenäs-Plate, L., Samuelsson, L., Tulinius, M. & Wentz, E. Autism spectrum conditons in myotonic dystrophy type 1: A study on 57 individuals with congenital and childhood forms. *Am. J. Med. Genet. Part B Neuropsychiatr. Genet.* **147B**, 918–926 (2008).
9. Phillips, M. F., Steer, H. M., Soldan, J. R., Wiles, C. M. & Harper, P. S. Daytime somnolence in myotonic dystrophy. *J. Neurol.* **246**, 275–282 (1999).
10. Helmrich, S. P., Ragland, D. R. & Paffenbarger, R. S. J. Prevention of non-insulin-dependent diabetes mellitus with physical activity. *Med. Sci. Sports Exerc.* **26**, 824–830 (1994).
11. Brook, J. D. *et al.* Molecular basis of myotonic dystrophy: expansion of a trinucleotide (CTG) repeat at the 3' end of a transcript encoding a protein kinase family member. *Cell* **68**, 799–808 (1992).
12. Cho, D. H. & Tapscott, S. J. Myotonic dystrophy: Emerging mechanisms for DM1 and DM2. *Biochim. Biophys. Acta - Mol. Basis Dis.* **1772**, 195–204 (2007).
13. Jaspert, A., Fahsold, R., Grehl, H. & Claus, D. Myotonic dystrophy: correlation of clinical symptoms with the size of the CTG trinucleotide repeat. *J. Neurol.* **242**, 99–104 (1995).
14. Sobczak, K., de Mezer, M., Michlewski, G., Krol, J. & Krzyzosiak, W. J. RNA structure of trinucleotide repeats associated with human neurological diseases. *Nucleic Acids Res.* **31**, 5469–5482 (2003).

15. Wheeler, T. M., Krym, M. C. & Thornton, C. A. Ribonuclear foci at the neuromuscular junction in myotonic dystrophy type 1. *Neuromuscul. Disord.* **17**, 242–247 (2007).
16. Yuan, Y. *et al.* Muscleblind-like 1 interacts with RNA hairpins in splicing target and pathogenic RNAs. *Nucleic Acids Res.* **35**, 5474–5486 (2007).
17. Koch, K. S. & Leffert, H. L. Giant hairpins formed by CUG repeats in myotonic dystrophy messenger RNAs might sterically block RNA export through nuclear pores. *J. Theor. Biol.* **192**, 505–514 (1998).
18. Timchenko, N. A. *et al.* RNA CUG repeats sequester CUGBP1 and alter protein levels and activity of CUGBP1. *J. Biol. Chem.* **276**, 7820–7826 (2001).
19. Philips, A. V, Timchenko, L. T. & Cooper, T. A. Disruption of splicing regulated by a CUG-binding protein in myotonic dystrophy. *Science* **280**, 737–741 (1998).
20. Jansen, G. *et al.* Abnormal myotonic dystrophy protein kinase levels produce only mild myopathy in mice. *Nat. Genet.* **13**, 316–324 (1996).
21. Wheeler, T. M., Lueck, J. D., Swanson, M. S., Dirksen, R. T. & Thornton, C. A. Correction of ClC-1 splicing eliminates chloride channelopathy and myotonia in mouse models of myotonic dystrophy. **117**, (2007).
22. Savkur, R. S., Philips, A. V & Cooper, T. A. Aberrant regulation of insulin receptor alternative splicing is associated with insulin resistance in myotonic dystrophy. *Nat. Genet.* **29**, 40–47 (2001).
23. Nakamori, M., Kimura, T., Fujimura, H., Takahashi, M. P. & Sakoda, S. Altered mRNA splicing of dystrophin in type 1 myotonic dystrophy. *Muscle Nerve* **36**, 251–257 (2007).
24. Dial, A. G., Ng, S. Y., Manta, A. & Ljubicic, V. The Role of AMPK in Neuromuscular Biology and Disease. *Trends Endocrinol. Metab.* **xx**, 1–13 (2018).
25. Kouki, T. *et al.* Low-dose metformin improves hyperglycaemia related to myotonic dystrophy. *Diabet. Med.* **22**, 346–347 (2005).
26. Laustriat, D. *et al.* In Vitro and In Vivo Modulation of Alternative Splicing by the Biguanide Metformin. *Mol. Ther. Nucleic Acids* **4**, e262 (2015).
27. Brockhoff, M. *et al.* Targeting deregulated AMPK / mTORC1 pathways improves muscle function in myotonic dystrophy type I. **127**, 549–563 (2017).
28. Hood, D. A., Uguccioni, G., Vainshtein, A. & D'souza, D. Mechanisms of exercise-induced mitochondrial biogenesis in skeletal muscle: implications for health and disease. *Compr. Physiol.* **1**, 1119–1134 (2011).
29. Ravel-chapuis, A., Al-rewashdy, A., Bélanger, G. & Jasmin, B. J. Pharmacological and physiological activation of AMPK improves the spliceopathy in DM1 mouse muscles. **27**, 3361–3376 (2018).
30. Manta, A. *et al.* Chronic exercise mitigates disease mechanisms and improves muscle function in myotonic dystrophy type 1 mice. **0**, 1–21 (2019).
31. Wright, N. C. *et al.* Aerobic walking in slowly progressive neuromuscular disease: Effect of a 12-week program. *Arch. Phys. Med. Rehabil.* **77**, 64–69 (1996).
32. Ørngreen, M. C., Olsen, D. B. & Vissing, J. Aerobic training in patients with myotonic dystrophy type 1. *Ann. Neurol.* **57**, 754–757 (2005).
33. Ng, S. Y., Manta, A. & Ljubicic, V. Exercise Biology of Neuromuscular Disorders. *Appl. Physiol. Nutr. Metab.* **1206**, apnm-2018-0229 (2018).
34. Tarnopolsky, M. A., Pearce, E., Smith, K. & Lach, B. Suction-modified Bergström muscle

biopsy technique: Experience with 13,500 procedures. *Muscle and Nerve* **43**, 717–725 (2011).
