# Supplementary material for: Aerobic exercise elicits clinical adaptations in myotonic dystrophy type 1 patients independently of pathophysiological changes
Source: J Clin Invest. 2022 May 16;132(10):e156125. doi: 10.1172/JCI156125 (PMC9106360; doi:10.1172/JCI156125)
Supplement: ICMJE disclosure forms [file jci-132-156125-s236.zip › COI_156123-JCI-CMED-RV-2/MAT_ COI_156125-JCI-CMED.pdf]

ICMJE DISCLOSURE FORM

Date: 2/8/2022

Your Name: Mark A Tarnopolsky

Manuscript Title: Aerobic exercise elicits clinical adaptations in myotonic dystrophy type 1 patients independent of pathophysiological changes.

Manuscript Number (if known): 156125-JCI-CMED-RV-2

In the interest of transparency, we ask you to disclose all relationships/activities/interests listed below that are related to the content of your manuscript. "Related" means any relation with for-profit or not-for-profit third parties whose interests may be affected by the content of the manuscript. Disclosure represents a commitment to transparency and does not necessarily indicate a bias. If you are in doubt about whether to list a relationship/activity/interest, it is preferable that you do so.

The author's relationships/activities/interests should be defined broadly. For example, if your manuscript pertains to the epidemiology of hypertension, you should declare all relationships with manufacturers of antihypertensive medication, even if that medication is not mentioned in the manuscript.

In item #1 below, report all support for the work reported in this manuscript without time limit. For all other items, the time frame for disclosure is the past 36 months.

		**Name all entities with whom you have this relationship or indicate none (add rows as needed)**	**Specifications/Comments (e.g., if payments were made to you or to your institution)**
**Time frame: Since the initial planning of the work**			
1	All support for the present manuscript (e.g., funding, provision of study materials, medical writing, article processing charges, etc.) No time limit for this item.	☐ None	
		CIHR Foundation grant to MAT (143325)	Grant in aid – supported some of the personnel and research costs for the current study.
		Neil and Leanne Petroff – donation to MAT through Hamilton Health Sciences Foundation.	Donation to MAT to support research in DM1 – supported research costs for the current study.
			Click the tab key to add additional rows.
**Time frame: past 36 months**			
**2**	Grants or contracts from any entity (if not indicated in item #1 above).	☒ **None**	
**3**	Royalties or licenses	☒ **None**	

		Name all entities with whom you have this relationship or indicate none (add rows as needed)	Specifications/Comments (e.g., if payments were made to you or to your institution)
**4**	Consulting fees	☐ **None**	
		Galapigos therapeutics.	I have received consulting fees for advice on Duchenne MD study trial design.
**5**	Payment or honoraria for lectures, presentations, speakers bureaus, manuscript writing or educational events	☐ **None**	
		Sanofi-Genzyme (2019-22)	Several lectures related to Pompe disease and hyperCKemia.
		Sanofi-Genzyme (2020-22)	Speaker bureau related to Pompe disease.
**6**	Payment for expert testimony	☐ **None**	
		Expert testimony – 2021-22 (CMPA)Sanofi	Case regarding spinal cord issue.
**7**	Support for attending meetings and/or travel	☒ **None**	
**8**	Patents planned, issued or pending	☐ **None**	
		Canadian Patent-3,050,823	Weight management patient to MAT.
		US patent – 11,167,001	Weight management patient to MAT.
**9**	Participation on a Data Safety Monitoring Board or Advisory Board	☐ **None**	
		Reneo Pharmaceuticals.	DSMB – PPAR gamma agonist for primary mitochondrial disease.
**10**	Leadership or fiduciary role in other board, society, committee or advocacy group, paid or unpaid	☒ **None**	

		Name all entities with whom you have this relationship or indicate none (add rows as needed)	Specifications/Comments (e.g., if payments were made to you or to your institution)
**11**	Stock or stock options	☐ **None**	
		Exerkine Corporation	I own stocks but have not received any payments from these.
**12**	Receipt of equipment, materials, drugs, medical writing, gifts or other services	☒ **None**	
**13**	Other financial or non-financial interests	☐ **None**	
		Exerkine Corporation	I am the founder and CEO. We have no DM1 patents or products at this point but may consider some in the future if the opportunity and/or concepts arise.
		Cora Therapeutics	I have warrants in exchange for scientific advice. The company goal is to develop nutraceuticals for radiation protection.

Please place an "X" next to the following statement to indicate your agreement:

☒ I certify that I have answered every question and have not altered the wording of any of the questions on this form.
